# *S. aureus* biofilm disruption using ultrasound and microbubbles: Influence of radiation force, bubble dynamics and biofilm growth conditions

**DOI:** 10.1016/j.bioflm.2025.100327

**Published:** 2025-10-25

**Authors:** Damien V.B. Batchelor, Anjali Lad, Kathryn L. Burr, Kristian Hollie, James R. McLaughlan, W. Bruce Turnbull, Jonathan A.T. Sandoe, Stephen D. Evans

**Affiliations:** a School of Physics and Astronomy, University of Leeds, Leeds, LS2 9JT, UK; b School of Chemistry and Astbury Centre for Structural Molecular Biology, University of Leeds, Leeds, LS2 9JT, UK; c School of Electronic and Electrical Engineering, University of Leeds, Leeds, LS2 9JT, UK; d Leeds Institute of Medical Research, University of Leeds, St James' University Hospital, Leeds, LS9 7TF, UK; e School of Medicine and Health, University of Leeds, Leeds, LS2 9JT, UK

## Abstract

*Staphylococcus aureus* is a human pathogen and a major cause of bloodstream infections, which can readily form biofilms on implanted medical devices. Here, we utilise a combination of lipid-shelled microbubbles (MBs) and ultrasound (US) to physically disperse the biofilm from the growth surface. The effects of two peak negative pressures (PNPs) and the direction of the acoustic radiation force (ARF) were evaluated. At 1.1 MHz, a clinically relevant frequency, and low PNP of 360 kPa, no significant biofilm dispersal occurred regardless of ultrasound (US) orientation. In contrast, at a high PNP of 2500 kPa, directing the ultrasound beam upward (US↑) pushed microbubbles (MBs) toward the biofilm, resulting in near-complete dispersal of the biofilm (94 ± 2 %) within the focal zone. Reversing direction to US↓, which pushes MBs away from the biofilm, reduced biofilm dispersal to 81 ± 3 %. Pre-treatment of the biofilm growth surface with fibrinogen or human plasma significantly altered the biofilm morphology and thickness, but did not affect the efficiency of ultrasound and microbubbles (US + MB)-mediated dispersal. Furthermore, multiple consecutive US + MB treatments could be applied to treat larger areas of biofilm without requiring MB replenishment between treatments. High-speed imaging was used to observe MB behaviour (e.g. translation and destruction) during US exposure. We revealed that the near instantaneous destruction of smaller MBs (∼1 μm) at high pressure did not induce significant biofilm dispersal and hypothesise that the translational motion of larger MBs (>10 μm) across the surface of the biofilm was the dominant mechanism behind biofilm dispersal.

## 1 Introduction

Approximately 25 % of the population is persistently colonised with the gram-positive bacterium *Staphylococcus aureus*, with the remaining 75 % intermittently or never colonised [1]. Whilst many of those colonised with *S. aureus* may never develop an infection, invasive medical procedures, such as the implant of medical devices (cardiac pacemakers, prosthetic heart valves or joints, urinary tract and central venous catheters), can provide an entrance point for pathogenic bacteria to enter the bloodstream [2]. *S. aureus* bloodstream infections have mortality rates of 20–40 % [3] and are a daily occurrence: *S. aureus* infections were the leading bacterial cause of death in 135 countries and associated with over 1.1 million deaths worldwide [4]. Implanted medical devices provide a surface to which bacteria can readily adhere and mature to form surface-attached biofilms [5] and *S. aureus* is adept at forming biofilm infections. Surface-attached biofilms consist of microbial communities encased in a self-produced extracellular polymeric substance (EPS), composed of proteins, carbohydrates, and extracellular DNA [5,6].

At the time of first clinical presentation, the presence of a *S. aureus* biofilm infection is usually not immediately apparent and initial treatment involves empirical antibiotics, followed by more specific, targeted antibiotic treatment once the type of infection is identified through microbiological cultures and other investigations [7]. The presence of EPS provides a physical and chemical barrier to the delivery of antibiotics, such that their susceptibility compared to planktonic bacteria is decreased by between 100- and 1000-fold [8], a contributing factor to treatment failure and the development of antimicrobial resistance. Further still, many *S. aureus* strains are also classified as antibiotic resistant. For example, methicillin resistance *S. aureus* (i.e. MRSA), which accounts for ∼10 % of all *S. aureus* infections [9] is resistant to the entire class of beta-lactam antibiotics (i.e. methicillin, penicillin) due to mutations of the penicillin-binding protein, PBP2a. As such, new treatment strategies are urgently required.

Microbubbles (MBs) are gas-cored bubbles, on the order of 1–10 μm in diameter, with a stabilising shell typically composed of lipid or polymer. MBs are widely used clinically as ultrasound (US) contrast agents as they can freely circulate through the vasculature, and their gas core provides a high acoustic impedance mismatch, generating ultrasound contrast [10,11]. When MBs are driven at their resonance frequency, which is within the clinical range for US imaging, contrast is further enhanced and MBs are regularly used to study blood flow in cardiac imaging [12]. The use of US is appealing due to its low cost, wide availability and non-ionising nature, whilst allowing for real-time imaging and good tissue penetration [13,14]. When exposed to an ultrasonic field MBs undergo volumetric oscillations, efficiently scattering the US waves, further enhancing image contrast [15,16]. MBs can also be targeted specifically to areas of interest through the attachment of targeting ligands, such as antibodies, to the shell, which shows promise for the detection of specific vascular biomarkers of disease (e.g. Vascular Endothelial Growth Factor Receptor 2,VEGFR-2, for the detection of new vasculature associated with fast growing cancers) [[17], [18], [19]]. The benefits of these oscillations extend not only to diagnostic applications, but also for therapy: oscillating MBs have been shown to locally exert shear stresses which can increase cell membrane permeability, enhancing drug uptake and treatment efficacy (sonoporation) [[20], [21], [22], [23]].

Recently, MBs have emerged as a potential tool to diagnose and treat biofilm infections. Firstly, MBs have been shown to be able to target and attach to biofilms either through electrostatic interactions [24], the incorporation of vancomycin directly into the bubble shell [25] or via ligand attachment such as lectins to target the EPS [26], or anti-body [26] and affimers [27] to target cell wall proteins. This opens the possibility to specifically locate the infection site *in vivo* through the use of US contrast imaging, as recently demonstrated using an *in vitro* model by Kouijzer et al. [25]. Additionally, in a process termed “sonobactericide” [28], MBs can be used to mechanically disrupt and disperse biofilms [29,30], with the aim to enhance the delivery of antibiotics either allowing for increased treatment effectiveness, or the ability to reduce the total antibiotic dose required. It should be noted that whilst US alone can act to disrupt biofilms [[31], [32], [33]], these studies typically require low frequency US (80–200 kHz), increasing the mechanical index and hence the likelihood of negative bio-effects such as tissue damage associated with transient cavitation [34,35].

Whilst it has been shown that MBs are able to physically disrupt biofilms, current studies have a few limitations. Firstly, many studies only utilise one US orientation, typically directing US such that MBs are forced towards the biofilm surface [27,30,36]. Whilst the use of targeted MBs may be able to ensure proximity of MBs to biofilms, it is not known how the influence of the direction of applied US, and hence associated acoustic radiation force (ARF), will have on biofilm dispersal. For example, in an intra-luminal catheter infection, the biofilm is associated with the entire lumen of the catheter and hence the ARF and MBs will interact differently with different areas of the infection, when exposed to unidirectional US.

Secondly, many studies consider MB interactions and biofilm removal across a small area (∼0.01 mm^2^), much lower than that typically associated with US beam (∼1 mm^2^). Whilst these studies can provide valuable insights into the interactions between MBs and biofilms, especially through the use of high-speed imaging [29], interactions on a larger scale within the entirety of the treated area are not well understood. Whilst some studies do assess biofilm removal over a larger area [36] (∼25 mm^2^), the interactions between MBs and biofilms and the mechanism of action are unknown. Further, there are variation in biofilm growth conditions between studies, such as growth medium (MHB [27], BHI [37,38], IMDM [25,39], DMEM [40,41]), growth time (24–96 h [25,[31], [32], [33],^42^]), static [36,42] or flow conditions [25,27,38], and surface pre-treatment (no treatment [31,32,43], fibronectin [36], fibrinogen [27], human plasma [25,30,38,41]) which have potential to influence biofilm morphology, and hence the ability of MB + US disruption [44].

In this work, we investigate the amount of biofilm disruption and removal as a function of radius from the central point of a focused US treatment beam (beam diameter ∼ 2 mm^2^) across biofilms grown on a microfluidic chip (length scale ∼ 10 mm^2^), through a combination of confocal fluorescence and high-speed microscopy. Firstly, we investigate how the direction of the acoustic radiation force (ARF), as well as US pressure, affect biofilm dispersal and MB behaviour within microfluidic devices. The behaviour of MBs during insonation, and hence the mechanism of biofilm removal, is then investigated using imaging across the whole microfluidic chip as well as high-speed imaging. We then assess whether pre-treatment of the biofilm growth surface with either fibrinogen or human plasma effects the magnitude MB-induced biofilm removal. Finally, we investigate whether repeat US treatments can be used on the same biofilm to increase the area of biofilm disruption, with or without replenishment of the MB solution.

## 2 Methods

### 2.1 Bacteria handing and culture

The S. *aureus* strain SH1000 [45] used throughout this study was stored as a frozen glycerol stock in a −80 °C freezer. The SH1000 stock was sub-cultured onto sterile horse blood agar plates (Thermo Fisher Scientific, Waltham, MA, USA) and incubated for 24 h, followed by storage at 4 °C. After inoculation, agar plates were used for culture for a maximum of 1 week. For liquid cultures, a single SH1000 colony was added to 10 mL of Muller Hinton Broth (MHB, Merck, Germany) and cultured overnight in an orbital shaker (37 °C, 200 RPM). Absorbance of the liquid culture at 600 nm (OD600) was measured in 96-well plates, and the dilution required to reach a final bacteria concentration of 1.5 × 10^8^ CFU/mL (0.5 McFarland Standard) was calculated. The culture was centrifuged in a 1 mL centrifuge tube (6000 g, 10 min), the supernatant removed, and the bacterial pellet resuspended in an appropriate volume of Dulbecco's Modified Eagle Medium (DMEM 41965, Gibco, US), prior to use to inoculate biofilm models.

### 2.2 Microfluidic biofilm models

Biofilms were grown in commercially available microfluidic devices (μ-Slide VI 0.4, Ibidi, Germany). Each microfluidic device consisted of 6 individual channels with a channel height of 0.4 mm, a length of 17 mm and a width of 3.8 mm. Biofilms were grown under a constant flow of DMEM at a volumetric flow rate of 40 μL/min. A schematic of the flow system is shown in Fig. S1a. A 50 mL syringe and syringe pump (PHD ULTRA, Harvard Apparatus, US) were used to control flow. The syringe was connected to a 250 mm section polypropylene tubing (inner diameter = 1/16″, outer diameter = 1/8″) using 1/16” barbed Luer female connectors. This section of tubing was connected in-line to an assembly of two 3-way Luer stopcocks, which in turn were attached to a 100 mm section of tubing, prior to connection to the microfluidic device. Interfacing to each channel was performed using elbow Luer connectors (10802, Ibidi, Germany). Each of the six channels were connected to its neighbouring channel via a daisy-chaining method using a 25 mm piece of tubing between each Luer connector, to increase throughput and ease of growth. A schematic of the daisy-chaining is shown in Fig. S1b. The final channel of the device was connected to a 2-way Luer stop-cock via a 100 mm length of tubing, followed by an additional 100 mm of tubing connected to the waste pot. Prior to use, the entire system was sterilised using 70 % EtOH for 30 min, followed by washing with DMEM. The system was inoculated by manual injection of 5 mL of a bacterial solution (1.5 × 10^8^ CFU/mL) through the first 3-way stopcock. The presence of the 2nd stopcock allowed the removal of any unwanted air bubbles introduced into the system at this stage. The system was left static for 2 h, with all stop-cocks in the closed position to prevent flow to allow adherence of biofilm to the microfluidic device. When the biofilm was desired to be formed on the upper face of the microfluidic channel, the microfluidic chip was inverted for the entirety of the culture process. After the 2-h static period, all stopcocks were opened and flow started.

#### 2.2.1 Surface pre-treatment

In some cases, the microfluidic chip was pre-coated with either fibrinogen from human plasma (F3879, Sigma Aldrich, US) or citrated mixed-pool human plasma (PS100-500-U1, TCS Biosciences, UK) for 2 h. Fibrinogen was prepared as previously described [46] to a working concentration of 1 mg/mL. The entire system was pre-sterilised with 70 % EtOH as before and then washed with DMEM. To prevent fibrinogen or plasma treatment of the tubing used during biofilm growth, the inlet tubing was disconnected from the first microfluidic channel and a syringe containing either fibrinogen or plasma was connected directly to this channel, then manually flushed through the ensure coating of all channels. This was left for 2 h before reattachment of the inlet tubing and washing with DMEM, followed by inoculation.

### 2.3 Confocal fluorescence imaging

Microfluidic chips were imaged using laser scanning confocal microscopy (Leica DMi8/SP8, Leica, Germany) to assess biofilm coverage before and after treatment. Biofilms were stained using SYTO9 (10 μM, 3 × 100 μL), a membrane permeable nucleic acid stain commonly used to stain live and dead gram-positive bacteria. Images were taken using a 488 nm laser with emission windows of 502–628 nm. Fluorescence and brightfield maps of each microfluidic channel were taken using the TileScan feature, consisting of multiple images (1024 × 1024 px) which were then combined to create the final image. Focus for each tile was achieved using a focus map, in which the focal point of 9 pre-defined points were chosen to create a map of focal points for the entirety of the TileScan image. A pin hole size of 3 Airy Units was used for TileScan imaging such to reduce the effect of any focus error associated with the focus map. Images were taken for each biofilm before and after treatment. To determine biofilm thickness and morphology under differing growth conditions, confocal z-stacks were taken at 1024 × 1024 px resolution, a z-step size of 1 μm, and pin hole size of 1 Airy Units.

#### 2.3.1 Confocal fluorescence biofilm removal analysis

A custom image analysis script was created using python and the OpenCV computer vision library to assess biofilm removal after MB + US treatment. Briefly, TileScan images were converted to binary via adaptive thresholding to determine biofilm coverage (Equation (1)). Biofilm coverage was then assessed as a function of radial distance and angle from the centre of the user-defined US treatment region. Biofilm removal was then calculated using Equation (2), such that biofilm coverage and removal was normalised to the biofilm before treatment.

Equation 1 $$\text{Biofilm}\;\text{Coverage}\;\left(\%\right)=100\;x\left[\frac{\text{Biofilm}\;\text{Area}}{\text{Total}\;\text{Area}}\right]$$

Equation 2 $$\text{Biofilm}\;\text{Removal}\;\left(\%\right)=100\;x\left[1-\frac{\text{Biofilm}\;{\text{Coverage}}_{\text{treated}}}{\text{Biofilm}\;{\text{Coverage}}_{\text{control}}}\right]$$

#### 2.3.2 Confocal fluorescence determination of biofilm thickness

To calculate biofilm thickness and roughness, confocal z-stack images of biofilms were acquired and analysed. A threshold was applied to each slice in the stack to determine the presence or absence of biofilm for each pixel. Biofilm thickness and roughness was determined as the average and standard deviation of the z position of the top surface of the biofilm.

### 2.4 Microbubble preparation and characterisation

An initial MB suspension was prepared using 95:5 M ratio of the lipids 1,2-dipalmitoyl-*sn*-glycero-3-phosphocholine (DPPC) and 1,2-distearoyl-*sn*-glycero-3-phosphoethanolamine-N-[methoxy(polyethylene glycol)-2000] (DSPE-PEG2000) to form the stabilising shell. Lipids were initially dissolved in 50:50 chloroform:methanol solution, and the solvent removed under nitrogen for ∼60 min, followed by vacuum desiccation overnight. The resultant lipid film was then rehydrated with PBS containing 1 % (v/v) glycerol, by stirring and heating at 55 °C for 20 min, to a final lipid concentration of 2 mg/mL. The lipid solution was then tip sonicated (10 % power, 20 kHz, 150 W, Sonifier 250, Branson, USA) for 40 min at 4 °C to produce small lipid vesicles (∼100 nm) [47]. This solution was then centrifuged at 17,000g for 30 min and aspirated, first to remove any titanium deposited during the tip sonication process and second to ensure the absence of any large lipid aggregates. To produce the initial bubble solution, 1 mL of vesicle solution was added to a 1.5 mL glass vial, and the solution and vial headspace was saturated with perfluorobutane (C_4_F_10_) gas, maintaining a gas pressure of 50 mbar for 2 min. Gas flow was controlled using a p-pump (Mitos P-pumps, Dolomite, UK) and a PC using the Dolomite Flow Control Centre. The vial lid was then replaced and sealed with parafilm, prior to mechanical agitation for 45 s (VialMix, Bristol Myers Squibb, USA). SonoVue MBs were prepared as per the manufacturer's instruction [48]. Briefly, the lyophilised powder was redispersed using 5 mL of sodium chloride solution, contained in a pre-filled syringe, using the Mini-Spike transfer system before vigorous manual shaking for 20 s. SonoVue MBs were used at their yield concentration.

Brightfield microscopy was used to determine the concentration of MBs. 30 μL of sample was introduced into a 50 μm depth chamber on a glass slide, and MBs allowed to rise for 5 min to ensure they were all in the same focal plane. An inverted microscope (90i, Nikon, Japan) was used to image the bubbles with a 40 × objective (NA = 0.6) and a CCD camera (DS-Fil 5Mega pixel, Nikon, Japan) was used to take 10 images for each sample. Images were then analysed using a custom MATLAB script to determine microbubble size and concentration [49].

### 2.5 Ultrasound system and treatment

A single element ultrasound (US) transducer with a central frequency of 1.1 MHz (H-102, Sonic Concepts, USA) was used for all US exposures. The transducer was connected to a +53 dB power amplifier (A150, E&I Ltd, USA) via an impedance matching circuit. The drive signal was generated using a computer-controlled function generator (TG5011A, Agilent, USA), providing sinusoidal bursts to the amplifier. A schematic of the US system is shown in Fig. S2. The peak free-field negative pressure of the transducer within the focal point of the ultrasound beam was determined using a needle hydrophone (0.2 mm, Precision Acoustics, Ltd., UK) calibrated by the National Physics Laboratory (Middlesex, UK). The hydrophone was mounted to a 3-dimensional translation stage (PT3/M, Thor Labs, Germany) equipped with micrometres engraved with 10 μm divisions. The location of the focal point of the ultrasound beam (i.e. maximum pressure) was determined by finding the local maximum voltage recorded from the hydrophone, observed using an oscilloscope (Waverunner, LeCroy, Chestnut Ridge, NY, USA). The recorded peak negative voltage was converted to pressure using a hydrophone sensitivity constant of 30 mV/MPa. Each US exposure consisted of the following parameters: drive frequency = 1.1 MHz, peak negative pressure = 360 or 2500 kPa, pulse repetition frequency = 1 kHz, duty cycle = 1 %, and total duration = 5 s.

The US transducer was coupled to microfluidic devices using a coupling cone containing degassed Milli-Q water. The top of the coupling cone was covered with an acoustically transparent membrane and held in place with an O-ring. This allowed the transducer to be inverted, and the US incident downwards onto the microfluidic chip when required. A thin water layer was added between the membrane and the microfluidic chip to ensure proper coupling. The microfluidic device was held within a 3D-printed holder that was designed to align the transducer with the longitudinal centre of the microfluidic channel. As each microfluidic device contains 6 channels, the holder enabled each channel to be manually moved into the acoustic focal point.

The focal region of the transducer (focal diameter = 1.3 mm, focal length = 10.21 mm, focal gain = 36.42) was aligned with the volumetric centre of each microfluidic channel. Each channel was individually filled with MBs (4 × 10^8^/mL) by pipetting 100 μL of sample directly into a reservoir and then withdrawing 100 μL from the opposing reservoir. This was repeated in triplicate to ensure the channel contained only MB solution. For US-only treated groups (i.e., no MBs), a PBS 1 % glycerol solution was used in place of the MB solution. Before treatment, the MBs were allowed to rise on the chip for 10 min (unless stated otherwise), ensuring they were in proximity to the biofilm.

### 2.6 High-speed imaging

To capture the US-induced behaviour of MBs on-chip, a high-speed camera (FastCam, Photron, Japan) was attached to an inverted brightfield microscope (Eclipse Ti2, Nikon, Japan) equipped with either a 4 × or 10× magnification objective. Additional light for high-speed imaging (1–30 kHz) was provided using an integrated light source within the central opening of the transducer, facilitated by a liquid light guide. The transducer was then coupled to the microfluidic chip from above, as described in Section 2.5. Particle tracking was performed using ImageJ/Fiji and the TrackMate plugin.

## 3 Results: microbubble-mediated biofilm removal in microfluidic models

### 3.1 Microfluidic biofilm growth

**Fig. 1:**
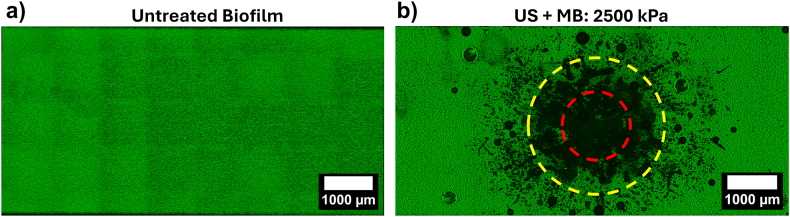
– Confocal microscopy images of *S. aureus* biofilms cultured on a microfluidic chip, with biofilm biomass shown in green (SYTO9 staining). a) An untreated biofilm after 24 h of growth. b) A biofilm after treatment with US (2500 kPa) and MBs (4 × 10^8^/mL) demonstrating removal of biomass within the focus of the US transducer. Red circle: 1 × focal radius (R_US_, 0.685 mm). Yellow circle: 2 × focal diameter (2R_US_, 1.37 mm). (For interpretation of the references to colour in this figure legend, the reader is referred to the Web version of this article.)

*S. aureus* biofilms were cultured in microfluidic devices, allowing for optical observation of biofilms before and after US + MB treatment, in addition to real-time observation of US-MB behaviour, as well as control of biofilm flow and growth conditions. Biofilms were cultured in mammalian cell culture media (DMEM) to mimic *in vivo* biofilm growth conditions (more so than standard culture media), which yielded consistent and homogeneous biofilm growth on-chip. During biofilm growth, a constant flow rate of 40 μL/min was used providing a continuous supply of media and nutrients. Use of flow and resultant shear stress (0.05 dyn/cm^2^) closer mimics that of the vascular system. Microfluidic devices consisted of 6 parallel channels daisy-chained together to provide higher throughput and allow testing of different conditions in parallel. Fig. 1a is an example of an untreated control biofilm, showing even coverage of biofilm across the entirety of the microfluidic device. It should be noted here that the tile pattern seen in these images is an artefact resulting from the stitching together of multiple images to create the final image, with a larger field of view. The MBs used in these experiments had an average size of 1.03 ± 0.03 μm, with a standard deviation of 0.87 ± 0.07 μm, and an average concentration of (3.4 ± 0.4) × 10^10^/mL (Fig. S3). Unless otherwise stated MBs were diluted to a concentration of 4 × 10^8^/mL and left to rise on-chip for 10 min to facilitate biofilm contact, before US exposures. After flotation and assuming all MBs have floated to the top of the microfluidic channel and hence in contact with the biofilm, the surface density of MBs was 0.16 MB/μm^2^.

### 3.2 Influence of ultrasound direction and pressure

In this section, we report the influence of US pressure and direction on the ability of both US and US + MBs to remove biofilms grown in microfluidic devices. All biofilms were cultured directly onto the hydrophobic microfluidic device substrate, with no surface pre-treatment. MBs were allowed to float towards and into contact with the biofilm, and as such, the direction of applied US would have influenced whether the acoustic radiation force (ARF) acted to push MBs either towards/into (US↑) or away (US↓) from the biofilm. In the case of US↑, microfluidic devices were inverted during culture such that the biofilms were grown on the top surface of the microfluidic channel, so MB flotation facilitated interaction with the biofilm, and US applied through the bottom substrate of the device. For US↓, the biofilms were grown on the bottom surface of the microfluidic channel, and the device then flipped before US treatment, facilitating MB flotation to the biofilm and ensuring the US beam path is identical to US↓ before reaching the microfluidic channel. This was performed so that the acoustic path is identical in both cases. A schematic of this is shown in Fig. S4. Two US peak negative pressures (PNPs) were investigated: 360 kPa and 2500 kPa, denoted as US(360) and US(2500). These two pressures were chosen as MB behaviour during insonation is pressure dependent and can be generalised into two types of behaviour. At low US driving pressures, MBs can undergo stable volumetric oscillations (stable cavitation), whilst increasing pressure above a certain threshold can induce MB collapse, implosion and destruction (inertial cavitation) [50]. Whilst the pressure threshold for inertial cavitation is dependent on multiple factors such as the US driving frequency, shell type, MB size (resonance frequency) and MB concentration, it is typically in the range of 600–1000 kPa, dependent on the size of the confining channel (e.g. a microfluidic chip) [51,52]. It should be noted that all pressures stated are the free-field pressure, and the *in-situ* pressure on the microfluidic chip may vary [53].

**Fig. 2:**
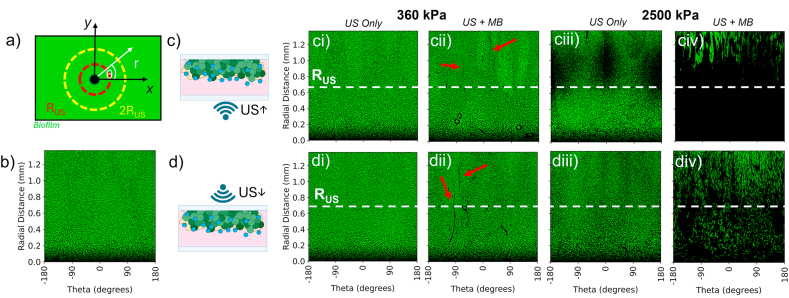
– a) Schematic showing the transformation of biofilm images from cartesian coordinates (x,y) into polar coordinates (r, θ), originating from the centre of the treatment area and extending to a maximum of twice the focal radius of the US beam (2R_US_). b) Example image of a control, non-treated biofilm after transformation into polar coordinates. Polar coordinate images of biofilms after (c) treatment with US from below (US↑) and d) after treatment with US incident from above (US↓) at PNPs of 360 kPa and 2500 kPa (US only or US + MBs). The edge of the US focal region, R_US_, is shown by a white dashed line. For PNP = 360 kPa conditions, areas of streak-like removal are highlighted by white arrows.

Initially, US was applied to the biofilms from below such that the ARF acted to push MBs towards the biofilm (US**↑**). An example image of a biofilm after treatment with US(2500)**↑** + MBs is shown in Fig. 1b, in which large amounts of biofilm were removed from a localised area within the US treated region. The US focal region is indicated by a red circle (1 × focal radius, R_US_, 0.685 mm) and a yellow circle (2R_US_). The US beam is radially symmetrical (Fig. S5), so images were transformed from cartesian coordinate system (x,y) to polar coordinates, to better convey biofilm removal. As such, these are shown as a function of radial distance, r, and angle, θ, originating from the centre of the treatment area and extending to a maximum of 2R_US_ (Fig. 2a). Fig. 2b shows a polar image of a control biofilm after no US treatment, demonstrating homogenous biofilm coverage across all radial distances. It should be noted that at small radial distances <0.1 mm, there are visible artefacts from the transformation due to low pixel count at these radii, which give rise to the dark band at the bottom of each image.

Fig. 2c shows biofilms after treatment with US**↑** only and US**↑** + MBs at a PNP of 360 kPa and 2500 kPa. For US(360)**↑** only, no change in biofilm morphology was observed compared to the untreated control. However, for US(360)**↑** + MBs, small streaks of biofilm were removed from the surface, radiating outwards. For US(2500)**↑** only, a band of biofilm removal was observed initially beginning at ∼ R_US_ and extending to ∼ 1.2 mm. For US(2500) **↑** + MB, the majority of biofilm within the focal region (i.e. < R_US_) was removed, above which streak-like radial patterns of biofilm removal were observed.

Next, the direction of the applied US was changed, such that the ARF was directed away from the biofilm surface. Polar images of biofilms after the application of US↓ are shown in Fig. 2d for PNPs of 360 and 2500 kPa respectively. For a US(360)↓, results were similar to that as for US(360)**↑**: little biofilm removal was observed for US(360)↓ only, whilst for US(360)↓ + MBs, small streaks of biofilm were removed. However, for US(2500)↓ results were markedly different: no obvious biofilm was removed for US(2500)↓ only and for US(2500)↓ + MBs, whilst large amounts of biofilm were dispersed, biofilm coverage still remained for distances < R_US_.

**Fig. 3:**
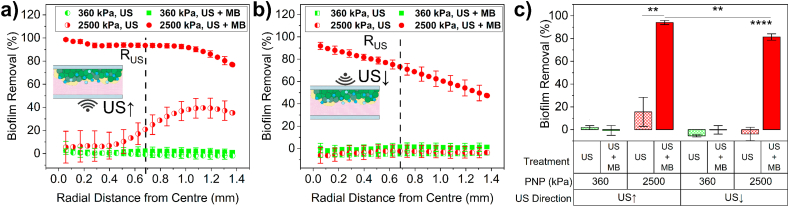
– Biofilm removal as a function of radial distance from the centre of the treatment area after treatment with (a) US**↑** and (b) US↓ at a PNP of either 360 kPa or 2500 kPa, for both US only and US + MBs. Dashed black line shows the location of the edge of the ultrasound focal point, R_US_. c) Total biofilm removal within R_US_ for both US orientations. Results show mean ± standard error over a minimum of 3 separate experiments. Stars signify varying levels of significance: p < 0.05, ∗; p < 0.01, ∗∗; p < 0.001, ∗∗∗, p < 0.0001, ∗∗∗∗.

These images were further analysed to quantify the percentage of biofilm removed, as a function of radial distance for both US**↑** (Fig. 3a) and US↓ (Fig. 3b). All data analysed is over a minimum of 3 separate experiments, and the errors shown represent the standard error across all repeats. For US(360)**↑** only, minimal biofilm removal was observed, whilst for US(360)**↑** + MB, small amounts of biofilm removal (<5 %) were observed only for distances > R_US_, correlating to the streak-like removal shown in polar images. Treatment with US(2500)**↑** only, initially showed little biofilm removal for distances < R_US_, which began to increase at ∼ R_US_ and reached a maximum of ∼40 % between ∼ 1.1 mm and 2R_US_. For US(2500)**↑** + MBs, biofilm removal was initially close to 100 % for distances <0.2 mm. This plateaued at ∼95 % removal between 0.2 and 0.9 mm, before decreasing to ∼80 % at 2R_US_.

Comparing to US↓ (Fig. 3b), minimal biofilm removal is observed for both US(360)↓ only and US(360)↓ + MBs. For US(2500)↓ only, no biofilm removal was observed at any radial distance, in contrast to US↑ where large amounts of removal were observed for distance > R_US_. For US(2500)↓ + MBs biofilm, large amounts of biofilms are still observed. Biofilm removal initially peaked at ∼80 % however this then decreased linearly with increasing radial distance to 55 % at R_US_ and to 20 % at 2R_US_, notably less than that observed for US↑.

To summarise biofilm removal for each treatment condition, these data were further analysed to determine total biofilm removal (TBR) within R_US_ (Fig. 3c). TBR within 2R_US_ is also shown in Fig. S6. At PNP = 360 kPa, for both US↑ and US↓, there was minimal TBR and no statistically significances (p > 0.05) were observed between US only and US + MB conditions for either US orientation. US(2500)↑+MBs removed 94 ± 2 % of biofilm within R_US_, compared to 16 ± 13 % for US(2500)↑ only, a statistically significant difference (∗∗, p < 0.01). Changing US orientation to US↓, for US(2500)↓ alone there was no biofilm removal within error observed within R_US_, whilst US(2500)↓ + MBs resulted in TBR of 81 ± 3 %. Comparing between the different US orientations, for the US(2500)↑ and US(2500)↓ conditions, there was no statistically significant differences in the amount of biofilm removed within R_US_. However, when considering removal within 2R_US_, the difference was significant (US(2500)↑: 26.4 ± 10.8 %, US(2500)↓: 3.5 ± 5.6 %). Comparing removal between US + MB conditions, treatment with US(2500)↑ was significantly higher than that for US(2500)↓ for both within R_US_ (94 ± 2 % vs 81 ± 3 %) and 2R_US_ (92 ± 2 vs 72 ± 5 %).

These findings demonstrate that there is a relationship between the PNP and TBR. At a PNP of 360 kPa, with or without MBs, minimal biofilm removal was observed for both US↑ and US↓. By increasing PNP to 2500 kPa, large amounts of biofilm (>80 %) were removed within the focal region of the transducer, regardless of US orientation. The direction of the applied US also had a significant influence on biofilm removal. Considering US(2500) only, for US↓ there was no observed biofilm removal. However, at the same PNP for US↑, a region of biofilm removal was observed between 0.6 and 1.4 mm, i.e. >R_US_, but little removal observed within the US focal region at distances < R_US_. Due to the high mechanical index of this treatment (MI ∼ 2.4) bulk cavitation in the water may be being induced and cause biofilm removal [54]. However, for this mechanism the area of biofilm removal would be expected to be maximum within the focal area of the ultrasound beam. We hypothesise that this may be due to US induced streaming within the fluid, near the biofilm-fluid interface due to the high focal gain of the US transducer (pressure focal gain = 36.42) and subsequent beam profile (Fig. S5).

For US + MB treatments, decrease in TBR for US↓ compared to US↑ may be associated with MB motion away from the biofilm due to the associated ARF, and hence MBs are not in close enough proximity to the biofilm to cause dispersal. Closer observation of biofilm removal (PNP = 2500 kPa) as a function of radial distance shows that for US_↑_ + MBs there is near total biofilm removal within the focal region of the US, however, at R_US_ biofilm removal starts to decrease with increasing radial distance, at which point streak-like patterns of biofilm removal become visible. Hence, it is possible that due to the high-focal gain of the transducer, the mechanism of biofilm removal with MBs is different within the focal region (<R_US_) compared to that outside (>R_US_).

#### 3.2.1 MB behaviour on-chip and mechanism of biofilm removal

To further investigate the behaviour of MBs on chip, and the mechanism of MB-induced biofilm removal, full images of the microfluidic devices were taken before and after US treatment at both PNP = 360 kPa and PNP = 2500 kPa (Fig. S7).

A subpopulation of MBs exceeding 10 μm in diameter (MB_>10 μm_) was identified on the microfluidic chip with a concentration of 1.9 ± 0.4 × 10^5^ MB/mL, representing approximately 0.04 % of the total MB population (4 × 10^8^ MB/mL). This proportion is noticeably lower than the 0.19 ± 0.06 % observed via post-production microscopy. The presence of MB > 10 μm is clinically relevant due to their potential to obstruct capillaries, contributing to micro-embolism, inflammation and thrombotic events [30]. Compared to clinically approved ultrasound contrast agents—Definity (1 %), SonoVue: 2 % Optison (5 %)—the proportion of large MBs in our formulation remains substantially lower. Further, our own characterisation of SonoVue MBs also revealed a population of large MBs, with an average on-chip size of 62 ± 18 μm, much larger than the MBs used in our study. The on-chip concentration of these larger MBs was determined to be (6.3 ± 0.7) x 10^4^/mL. However, the yield of SonoVue is known to be ∼2 × 10^8^/mL [48], 2 × lower than the MB concentration we have used in our studies (4 x 10^8^/mL). Accounting for this by multiplying by 2, the equivalent concentration of large SonoVue MBs was (1.3 ± 0.2) × 10^5^/mL, which is comparable to our MBs (Fig. S8). These findings suggest that the MBs used in this study pose no greater embolic risk than those currently in clinical use. 10.13039/100014337Furthermore, similar populations of MB > 10 μm have been reported in other MB-biofilm studies [9,14,15,32], supporting the broader relevance of these observations.

Treatment with US at 360 kPa appeared to have no effect on MB_>10 μm_. Before US treatment MB_>10 μm_ size was 38 ± 12 μm, expressed as modal size ±standard deviation, and after US, MB_>10 μm_ diameter was 38 ± 18 μm. Further, there was no change in MB_>10 μm_ concentration (before US: 1.6 ± 0.6) × 10^5^/mL, after US: 1.4 ± 0.5) × 10^5^/mL). Treatment with US at 2500 kPa however, did influence MB_>10 μm_. The MBs that were initially positioned within the focal region of the US, had assembled in a circle with a radius equal to the radius of the focal region, R_US_. The average size of MBs_>10μm_ on-chip had also increased from 36 ± 13 μm before US to 47 ± 21 μm after US, whilst MB_>10 μm_ concentration decreased from (2.0 ± 0.6) × 10^5^/mL to (1.2 ± 0.3) × 10^5^/mL, suggesting possible coalescence.

As the average size of the MB populations inoculated onto the chips in this study was ∼1 μm, automated characterisation of these MBs proved challenging due to the resolution of the imaging system (0.81 μm/pixel). However, manual inspection of the individual TileScan images (Fig. S9a) showed that these MBs_<10 μm_ were visible on-chip, appearing as a speckle-like background in the bright-field images. At PNP = 360 kPa, the presence of this background was removed within the US focal area, whilst outside the focal region the occurrence of this background remained. At a PNP of 2500 kPa, it appeared that the speckle pattern was reduced across the entire chip. To quantify this, the change in background intensity across the TileScan image after US treatment was determined (Supplementary Methods 1, Fig. S9b), whereby an increase in intensity correlated with removal of MBs. At PNP = 360 kPa, the change in background intensity peaked at ∼10 %, localised to a region ±2 mm from the centre of the US-treated region, suggesting that MB < 10 _μm_ are affected by US within a localised area. At PNP = 2500 kPa the change in background intensity appeared near uniform over the whole chip (±4 mm), at ∼ 35 %, and as such is interacting with small MBs across the entirety of the chip.

High-speed imaging was used to observe MB behaviour during insonation. The imaging system was capable of imaging frequencies of ∼10^4^ Hz, able to observe US-induced MB motion and translation (PRF = 10^3^ Hz) but unable to observe individual MB oscillations (driving frequency ∼ 10^6^ Hz). Here, only conditions of US↓ could be investigated due to physical limitations of the microscopy system. For each PNP, videos were acquired at varying magnification and frame rate, to capture MB behaviour over a range of length scales.

**Fig. 4:**
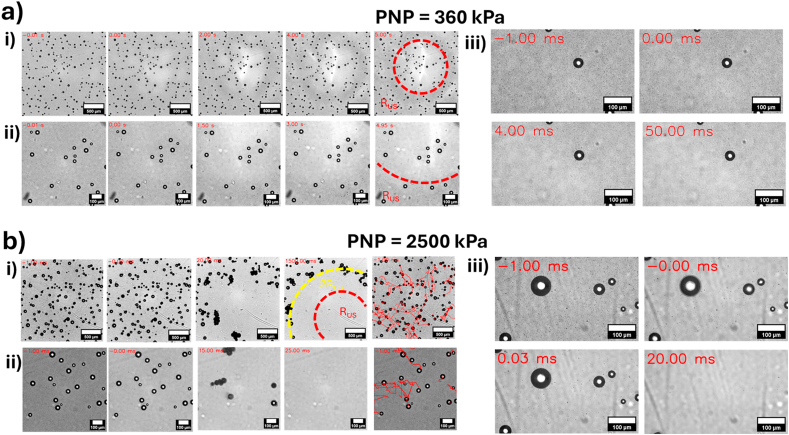
– Frames taken from high-speed videos of MB motion during insonation at (a) PNP of 360 kPa and (b) PNP of 2500 kPa. For PNP = 2500 kPa, particle tracks are showing as red lines in the last panel of (i) and (ii). Time stamps are shown in the top left corner of each frame in red. i) Wide field of view images (2166 μm × 2166 μm) in which behaviour throughout the entire focal area of the US beam (R_US_, red circle) can be observed. ii) Increased magnification images with a smaller field of view (866× 866 μm) at the edge of the focal region (red dashed circle). In the case of PNP = 2500 kPa, the final frame shows particle tracks overlayed on-top of the original image. iii) Zoomed in images (541 × 318 μm) taken within the focal region. (For interpretation of the references to colour in this figure legend, the reader is referred to the Web version of this article.)

Fig. 4 shows timestamped images from high-speed observation of MBs after treatment with PNP = 360 kPa (Fig. 4a) and PNP = 2500 kPa (Fig. 4b). Full high-speed videos are included in the Supplementary Material. At PNP = 360 kPa, no movement or destruction of MB > 10 μ_m_ was observed throughout the total duration of treatment (5 s). It is possible that these MBs were undergoing volumetric oscillations, which were not observable at the imaging frequency of ∼10^4^ Hz. However, US did influence smaller MBs. In Fig. 4ai, in which an area greater than that of the US focal region was observed, the image initially had a dark, grey background, potentially due to smaller MBs. Over the course of the full US exposure, this background began to disappear, radiating outwards from the centre of the image. As this system utilises transmitted light for imaging, this removal of background and localised increase in image brightness may correlate to the removal of small MBs from within this area. The diameter of this area of removed background also corresponded to that of R_US_. Images taken at an increased magnification at the edge of the focal region (Fig. 4aii) showed a somewhat similar trend. Initially small MBs were seen as a speckle pattern in the background of the image. Throughout US exposure, the large MBs remained undisturbed, and the speckle pattern began to decrease, radiating from the top-right corner of the image. At t = 4.95 s, the maximum duration of video acquisition, the radius of this area also appeared to correlate to R_US_. Additionally, small MBs could be seen to start clustering and coalescing to form larger MBs at a radius ∼ R_US_. Fig. 4aii shows images taken within the focal region of the US beam, over a smaller field of view, and showing smaller timesteps between each frame (∼10^−3^s), Again, the larger MB was unaffected by the US trigger, but smaller MBs began to disappear from the field of view over ∼ 50 ms. Results for biofilm dispersal shown in Fig. 3 at PNP = 360 kPa showed that there was no observed biofilm dispersal for radial distances < R_US_, but that small streaks of biofilm removal were found at distances ∼ R_US._ This is likely caused by the formation of either clusters of multiple MBs, or larger MBs formed from coalescence of smaller MBs, and their subsequent movement across the surface of the biofilm. Whilst we are unable to observe the exact acoustic behaviour of MBs, we hypothesise that at PNP = 360 kPa, MBs within the US focal region are likely dissolving over time due to instability caused by their US induced oscillations. Further, in the case of US↓, MBs are forced out of the imaging focal plane by the ARF and hence these MBs do not significantly contribute to biofilm dispersal.

At PNP = 2500 kPa, both larger and smaller MBs were influenced by the application of US. Assessment over a large field of view (Fig. 4bi) showed near instant removal of smaller MBs after the first US pulse, across the entirety of the image. Larger MBs initially remained unaffected, but prolonged US exposure (>10 ms, 10 pulses) induced in-plane motion in larger MBs which could also be observed interacting and coalescing with each other. By the end of the observable period (1500 ms), all MBs had vacated the area within 2R_US_ of the US beam and some MBs had assembled around the edge of this region (yellow circle), similar to Fig. S7. The translational motion of these larger MBs is also shown via image-tracking paths shown in the last panels of Fig. 4bi. A similar effect was observed over a small field of view (Fig. 4bii): a near instantaneous removal of the background of small MBs, followed by motion of larger MBs which moved out of the field of view. Observation of smaller MBs (Fig. 4biii) also showed near instantaneous removal of smaller MBs, followed by translation of larger MBs. This sudden disappearance of smaller MBs (i.e. within 1 US pulse, 1 ms), combined with the high PNP (2500 kPa), suggests that they may be experiencing inertial cavitation. However, there is also the possibility they are simply being pushed out of the optical imaging plane. We also observed that a PNP of 2500 kPa was capable of inducing motion of larger MBs within the focal region of the US beam. As this was the area in which the majority of biofilm removal was observed, we hypothesise that the larger MBs play a key role in biofilm removal.

### 3.3 Surface treatment for biofilm growth

Previously, we have shown that >90 % biofilm can be removed by US + MB treatment(Section 3.2). However, these biofilms were cultured on a bare, un-treated polymer surface. Pre-treatment of the biofilm growth surface is commonly used in *in vitro* models to aid bacterial attachment, as well as provide a closer mimic to those found *in vivo*. When medical devices are inserted *in vivo* they are rapidly coated by proteins contained within human plasma, which in turn influences the binding capability of *S. aureus* to the surface of the device [55]. Human plasma contains a variety of proteins, with the most abundant being albumin, globulins and fibrinogen. Of these, fibrinogen is of special interest as *S. aureus* expresses numerous fibrinogen binding proteins, which are capable of “hijacking” the host fibrinogen to form a fibrinogen/fibrin ‘shield’, protecting the bacteria from phagocytosis [44,56,57]. Here, we investigate if pre-treatment of the biofilm growth surface with either fibrinogen or human plasma influenced the resultant biofilm morphology and in turn if this affected the ability of US + MBs to disrupt the biofilm.

#### 3.3.1 Change in biofilm morphology

Biofilms were grown in microfluidic devices, on surfaces either with control conditions(no pre-treatment, akin to those in section 3.2), or after treatment for 2 h with either fibrinogen human plasma, prior to inoculate and growth under flow (Q = 40 μL/min). The fibrinogen concentration (1 mg/mL) was chosen as it is similar to that found in blood plasma [58,59], and would be expected to fully coat the surface after a 2-h incubation period [60].

**Figs. 5:**
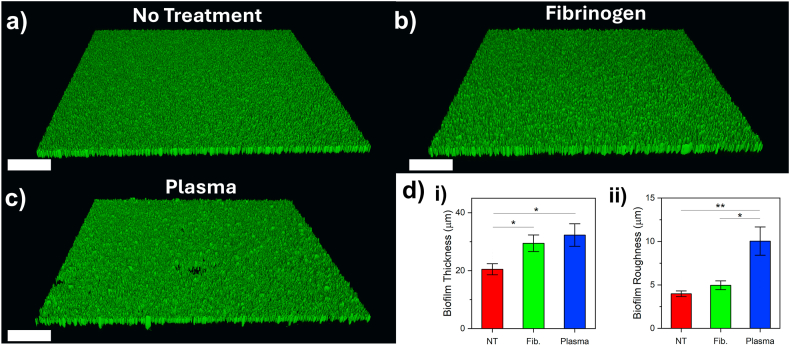
3D renders of confocal z-stack images showing biofilms grown after a) Control (no surface treatment), b) pre-treatment with fibrinogen and c) pre-treatment with plasma. Scale bar = 200 μm. Each image shows a 1162.5 x 1162.5 μm region of the biofilm. d) Corresponding biofilm thickness (i) and roughness (ii) after each pre-treatment condition. All experiments consisted of N = 3, separate experiments. Stars signify varying levels of significance: p < 0.05, ∗; p < 0.01, ∗∗.

For each biofilm treatment group, z-stacks were taken and rendered into 3D to observe changes in morphology. Confocal z-stack images of each type of biofilm are shown in Fig. 5 (scale bar = 200 μm). Control biofilms without any pre-treatment (Fig. 5a) had a smooth surface, whilst pre-treatment with fibrinogen appeared to create biofilms with increased surface roughness (Fig. 5b). Pre-treatment with human plasma (Fig. 5c) influenced biofilm morphology further still, with the presence of mushroom-like structures. Confocal z-stacks were subsequently analysed to determine biofilm thickness (Fig. 5di) and biofilm roughness (Fig. 5dii). Here, thickness was defined as the average height of the biofilm surface, and roughness as the standard deviation of this height across the entire biofilm. Control, no treatment biofilms were found to have an average thickness of 20 ± 2 μm. Pre-treatment of the growth surface increased biofilm thickness to 29 ± 3 μm and 32 ± 4 μm for fibrinogen and plasma treated biofilms respectively, both statistically significant increases (∗, p < 0.05) compared to no treatment controls. Surface treatment also increased the roughness of the biofilm: no treatment biofilms had a roughness of 4 ± 0.3 μm, which increased slightly with fibrinogen treatment (5 ± 0.5 μm) although this was found to be not statistically significant. Plasma treatment greatly increased biofilm roughness to 10 ± 1.6 μm, which was significant compared to both no treatment (∗∗, p < 0.01) and fibrinogen treatment (∗, p < 0.05).

#### 3.3.2 Analysis of US and MB mediated removal of fibrinogen and plasma surface treated biofilms

**Fig. 6:**
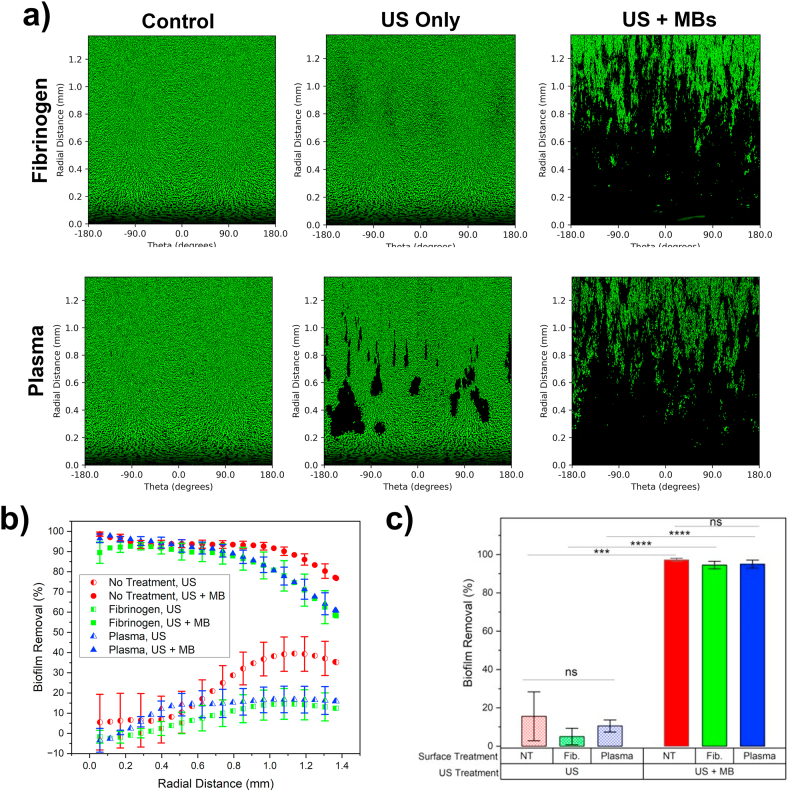
– a) Polar coordinate images of biofilms grown after pre-treatment of the growth surface with either fibrinogen or human plasma, showing biofilms after either no treatment (control), US only (PNP = 2500 kPa) or US + MBs (PNP = 2500 kPa, MB concentration = 4 × 10^8^/mL. b) Percentage of biofilm removal for each biofilm pre-treatment group (no treatment, fibrinogen, plasma) after treatment with either US only or US + MB as a function of radial distance from the centre of the US region. c) Total biofilm removal within R_US_ for each condition (no treatment = NT, fibrinogen = Fib.).

Next, biofilms were treated with US only and US + MBs (4 × 10^8^/mL) and biofilm removal compared between each pre-treatment condition. A PNP of 2500 kPa was chosen, and US treatment from below (US↑) as this was found previously to be most effective in biofilm removal. Fig. 6a shows polar images of biofilms grown after surface pre-treatment with either fibrinogen or human plasma, and either after no US treatment (control), US only or US + MBs. For fibrinogen treated biofilms, polar images were similar to that shown for control, non-treated biofilms (Fig. 2): for US only, areas of removal were seen at ∼ R_US_ and for US + MB the majority of biofilm is removed for distance < R_US_ and biofilm density increased for distances above this. For plasma biofilms treated with US only, large, discrete chunks of biofilm were removed. It should be noted that whilst this pattern was observed in 100 % of plasma biofilms, it was also noticed in 33 % (2 out of 6) experiments of fibrinogen US only treatments. For US + MB treatment there was still a majority of biofilm removal for < R_US_, and again density of biofilm increased for distances > R_US_, similar to that for fibrinogen treated biofilms. Next, biofilm removal as a function of distance was analysed, and compared to that of control biofilms with no pre-treatment, previously presented in Section 3.2. US only treatment on fibrinogen biofilms showed a similar trend to that of control no-treatment biofilms, but to a lesser extent: initially there was little biofilm removal which then increased to a maximum of ∼10 % for distances > R_US_, less than the 40 % observed for no treatment. Similarly, US + MB treatment followed a similar trend to the no treatment condition with ∼90 % of biofilm consistently removed for < R_US_. However, for distances > R_US_, the proportion of biofilm removed was noticeably less, and decreased from 89 % at 0.8 mm to 59 % at 2R_US_, compared to a decrease from 93 % to 77 % for no treatment biofilms. For the plasma treated biofilms, biofilm removal increased up to a peak removal of 14 % at a radial distance of 0.5 mm, maintain constant biofilm removal up to 2R_US_. For US + MB treatment, plasma biofilms followed a trend near identical to that for fibrinogen treated biofilms.

When considering the total biofilm removal within R_US_, it was found that for US only there was no statistically significant difference between TBR for control, no treatment (15 ± 13 %), fibrinogen (5 ± 4 %) or plasma (11 ± 3 %). This was also true for the US + MB treatment condition (control; 92 ± 2 %, fibrinogen; 85 ± 4 %, plasma; 95 ± 2 %). The increase in biofilm removal between US and US + MB treatment for each pre-treatment condition was found to be statistically significant (control; p < 0.001, ∗∗∗, fibrinogen and plasma; p < 0.001, ∗∗∗∗). Similar results were also found when considering removal within 2R_US_ (Fig. S10). As such, when treated with the combination of US + MB at a PNP of 2500 kPa, ∼90 % of the biofilm within the US focal region was removed, and the biofilm thickness and morphology prior to treatment had no effect on the total amount of biofilm removed.

### 3.4 Repeated US + MB treatment of biofilms

**Fig. 7:**
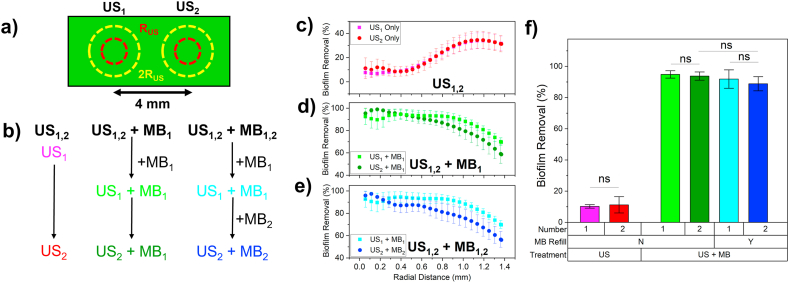
– a) Schematic showing two separate US treatments (US_1_ and US_2_) separated by 4 mm on the microfluidic device. Red circle = R_US_, Yellow Circle = 2 R_US_. b) Workflow of each treatment type for two treatments of US only (US_1,2_), US and MBs with no MB replacement (US_1,2_ + MB_1_) and US and MBs with MB replacement between US treatments (US_1,2_ + MB_1,2_). Biofilm removal as a function of radial distance for (b) US_1,2_, (c) US_1,2_ + MB_1_ and (d) US_1,2_ + MB_1,2_. e) Total biofilm removal within US focal region for each treatment condition. (For interpretation of the references to colour in this figure legend, the reader is referred to the Web version of this article.)

Whilst US + MBs could be seen to nearly entirely disperse biofilms from within the focal region of the US beam, this area was localised to within ∼ 2R_US_, corresponding to a total area of ∼6 mm^2^. For the treatment to be of use in a clinical setting, a larger area of biofilm would need to be treated and dispersed, which could potentially be performed by multiple applications of US in different regions. As such, we investigated whether US + MBs could be used to disperse biofilms across two separate areas on the microfluidic device. The microfluidic chip was treated twice with US, with a distance of ∼4 mm between US treatments, ensuring no overlap between the 2R_US_ regions and allowing for independent analysis of each US treatment (Fig. 7a). As the US treatment (PNP = 2500 kPa) appeared to interact with MBs over the full length of the microfluidic chip (at least ± 4 mm from the centre of R_US_, Fig. S9) we compared biofilm dispersal between the first US treatment (US_1_) and second US treatment (US_2_) using a single administration of MBs added before US_1_ (US_1,2_+MB_1_), compared to replenishment between US_1_ and US_2_ with fresh MB sample (US_1,2_+MB_2_). A timeline for each treatment condition is shown in Fig. 7b. For US_1,2_ only and US_1,2_ + MB_1_, the two US treatments were applied immediately after each other, whilst for US_1,2_+MB_1,2_ fresh MBs were added to the chip and allowed 10 min to rise before US_2_.

Results for biofilm removal as a function of radial distance are shown in Fig. 7c, d and e for US_1,2_, US_1,2_ + MB_1_ and US_1,2_ + MB_1,2_ conditions respectively. TBR within R_US_ for each condition is shown in Fig. 7f. For US_1,2_ only (Fig. 7c) biofilm removal was near identical between US_1_ and US_2,_ and following a similar trend to results previously shown in Fig. 3a for US↑ only. For US_1,2_ + MB_1_, biofilm removal was the same between the first US treatment (US_1_ + MB_1_) and second US treatment (US_2_ + MB_1_) for distances less than R_US_ (0.685 mm), and as such the difference of TBR within the focal region (Fig. 7f) was deemed insignificant (US_1_ + MB_1_ = 95 ± 2 %, US_2_ + MB_1_ = 94 ± 3 %). For distances outside the focal region (>R_US_) biofilm removal for US_2_ + MB_1_ was consistently ∼10 % lower than that for US_1_ + MB_1_. Although the underlying cause remains unclear, this trend is also observed under US_1,2_ + MB_1,2_ conditions. When MBs were replenished between US treatments (US_1,2_ + MB_1,2_), biofilm removal was slightly decreased by ∼ 6 % within the focal region (<R_US_), with this difference increased to ∼ 12 % outside the focal region (>R_US_). However, it was found there was non-significant difference in total biofilm removal within the focal region between US_1_ + MB_1_ (92 ± 6 %) and US_2_ + MB_2_ (89 ± 5 %).

Similarly, the difference between US_2_ + MB_1_ (94 ± 3 %) and US_2_ + MB_2_ (89 ± 5 %) was also deemed insignificant. Thus, US combined with MB effectively removed biofilm across multiple regions, with minimal variation in removal efficiency regardless of whether MBs were replenished between US treatments. To further understand this behaviour, full TileScan images were taken of the whole microfluidic chip (Fig. S11). For US_1_ in both treatment plans (US1,2 + MB1 and US1,2, + MB1,2) MB > 10 μm situated within the focal region of US1 were displaced, consistent with our previous findings. MBs situated elsewhere on-chip were also affected by the application of US1, with an increase in MB > 10 μm mean size from 16.1 ± 0.2 μm to 21.1 ± 0.4 μm, concurrent with a decrease in concentration from (1.64 ± 0.10) × 105/mL to (1.27 ± 0.11) × 105/mL, suggesting possible coalescence of the larger MBs, similar to that observed in Fig. 4. For the case of US1,2, + MB1,2, the addition of fresh MBs (+MB2) increased the MB > 10 μm concentration to (1.92 ± 0.20) × 105/mL. The mean size of MB > 10 μm also decreased to 16.5 ± 0.6 μm, suggesting many of the larger MBs had been removed in the washing process and replaced by smaller, fresh MBs. Following application of US2, again, MB > 10 μm within the focal region were displaced for both US1,2 + MB1 and US1,2, + MB1,2. The behaviour of smaller MBs analysed separately, similar to Section 3.2.1 and is shown in Fig. S12. For US1,2 + MB1, the application of US1 affected MBs throughout the entire observable area of the chip, indicated by a near uniform increase in background intensity of the image by ∼35 %. However, for distances >4 mm from the focal region, this began to decrease to ∼30 %, suggesting beyond this distance the concentration of small MBs begins to increase. The application of US2 at x = 4 mm increased the change in background intensity to approximately 35 % across the entire chip, effectively removing any remaining small MBs in this region. In the US1,2 + MB1,2 condition, similar results were initially observed: US1 changed the background intensity by ∼30 % uniformly across the chip. Addition of MBs (+MB2) decreased this change to ∼10 % which was then reversed by the application of US2, back to ∼30 %.

## 4 Discussion

This work revealed the pressure and directional dependence on US-mediated biofilm dispersal. Two US PNPs were investigated, 360 kPa and 2500 kPa, as well as the orientation of the applied US (either towards [US↑] or away from [US↓] the biofilm). At PNP = 360 kPa, quantitative analysis revealed minimal biofilm was dispersed for both US only and for US + MB conditions, regardless of the US direction. This result is somewhat surprising as a PNP of 360 kPa is similar to those used in previous studies in which biofilm was successfully dispersed (100–500 kPa [25,29,36,61]). Predicted wall shear stresses generated by a stably oscillating MB typically range from 1 to 20 kPa [62,63], which far exceed the reported thresholds for bacterial detachment and biofilm removal (0.01–100 Pa) [[64], [65], [66]]). It should be noted that MB behaviour will also be dependent on the relationship between US driving frequency and MB resonance frequency (itself dependent on MB size and shell parameters). Furthermore, most studies investigating shear effects on biofilms employ bulk fluid flow over much longer timescales (minutes to hours) compared to the relatively short exposure times typical of MB treatment (typically seconds). Previous studies also only typically consider a smaller area of biofilm (∼0.01 mm^2^) and at higher resolution in which smaller disruption to the biofilm may be observable. In this study, we are primarily interested in the US + MB mediated disruption over larger areas (∼1 mm^2^) and as such, with direct clinical relevance.

Brightfield microscopy and high-speed imaging revealed that MBs_>10μm_ were unaffected by US at PNP = 360 kPa. However, smaller MBs within the focal region of the US disappeared over ∼50 ms (i.e. 50 pulses), either dissolving or being pushed out of the imaging focal plane by the ARF (in the case of US↓). Whilst biofilm removal within R_US_ was deemed not statistically significantly different between US only and US + MB, continued insonation caused small MBs at ∼ R_US_ to coalesce to form larger MBs and MB clusters, similar to those shown in other studies [29,36]. These MBs were then observed translating in a radial direction, and correlated with small streaks of biofilm removal (white arrows, Fig. 2). This suggests that biofilm disruption may be induced by clusters of MBs, rather than individual MBs, potentially due to the non-linear relationship between MB size and the shear stress they generate [67].

Treatment of MBs at a PNP of 2500 kPa did disperse the vast majority of biofilm within both R_US_ (US↑: 94 %, US↓: 81 %) and 2R_US_ (US↑: 90 %, US↓: 70 %), also showing dependence on the orientation of the applied US. Whilst not directly observed here, the ARF would be expected to push MBs in the direction of propagation of the US beam [68] and correlates with a decrease in biofilm dispersal for US↓. This is also in line with work from Caskey et al. in which MBs were found to tunnel into a gel phantom exclusively on the distal side of the US transducer [69]. In our study, this dependence on US orientation is further highlighted by the US(2500)↑ only condition, where ∼ 30 % of biofilm was removed within 2R_US_, whereas for US(2500)↓, no biofilm removal was observed. Further, analysis of biofilm dispersal as a function of radial distance from the centre of the US beam allowed additional insight into the mechanism of removal, i.e. for (2500)↑ only, biofilm removal for distances < R_US_ peaked at ∼ 15 %, increasing to a maximum of ∼40 % for distances > R_US_, potentially due to US induced streaming. Assuming a peak acoustic intensity, I, of approximately 200 W/cm^2^ for our system (Methods S2) [70] we predict for the US(2500)↑ only condition induces wall stresses to be on the order of 0.1 Pa ([71]) which falls within the range required for shear-induced bacterial detachment (0.01–100 Pa).

Biofilms were cultured on surfaces with different pre-treatments. Pre-treatment with fibrinogen or human plasma both led to increased biofilm thickness and surface-roughness but had little effect on the effectiveness of the US + MB treatment. It should be noted here that biofilms were cultured for 24 h, and biofilm maturity is associated with increased tolerance to antibiotic treatments [72], which in addition, may further influence their mechanical properties. Further, differing *S. aureus* strains have been shown to adhere to surface with varying strengths [73], and hence future direction should involve the treatment of biofilms with increased maturity and a range of *S. aureus* strains.

High-speed observation of MBs insonated at 2500 kPa showed that all small MBs were removed from the field of view within a single US pulse (1 ms), potentially due to either inertial cavitation or rapid translation out of the imaging focal plane, faster than the observable frequency of the high-speed imaging system. However, it is likely the former as small MBs also disappeared with both US↓ and US↑ (Fig. S7).

Additional imaging of the entire chip before and after US revealed that the majority of small MBs on the chip were also removed. After ∼20 US pulses (20 ms), MBs_>10μm_ began to migrate away from the centre of the US beam, arranging in a circle of radius ∼ 2R_US_, whilst larger MBs situated off-centre remained unaffected. This finding is consistent with the motion and clustering of these larger MBs, suggesting that they are primarily responsible for the majority of biofilm dispersal. This is in line with Lattwein et al. [29], which reported that an increase in MB clustering and coalescence corresponded to increased biofilm dispersal (mean MB size = 4 μm, distribution range of 1–30 μm). As the activity of the larger MBs appeared to stabilize after ∼150 ms, the total duration of the US treatment (5 s) may be reduced, hence mitigating the risk of any unwanted bioeffects, such as off-focus cavitation or heating.

Step and repeat US + MB treatment successfully removed biofilm across the surface, showing promise for treatment of larger area infections. e.g. for treatment of the entire lumen of a central venous catheter.

It was shown that small MBs were removed from the entirety of the chip after the application of the first US treatment, whereas large MBs remained relatively unaffected. Interestingly, we found that there was no difference in biofilm dispersal regardless of whether the MB solution was replenished between US treatments, further reinforcing our hypothesis that large MBs dominate the biofilm dispersal process. Here, the concentration of MBs was fixed at 4 × 10^8^/mL, similar to those used in previous studies [[31], [32], [33],^36^] and those used clinically for diagnostic purposes [49]. As the relationship between MB cavitation threshold (stable and inertial) with MB concentration is non-linear, due to complex bubble-bubble interactions during insonation [74,75] there may exist an optimal combination of PNP and MB concentration for biofilm dispersal, whilst reducing unwanted US only effects. As the mean size of the MB population used here was relatively small (∼1 μm) and polydisperse, there would be merit in the use of monodisperse MBs, as their size can be tuned such that their resonance frequency matches that of the driving US [76], as well as their larger size in general lending itself to increased biofilm dispersal [67].

Further, the MBs study presented here relied on flotation of the MBs to be in proximity to the biofilm, which may be unrealistic in a clinical setting. However, this is somewhat of an approximation of the use of MBs targeted to the biofilm. Recently we developed MBs specifically targeted to *S. aureus* biofilms, capable of a bound MB density of ∼1.4 × 10^3^ MB/mm^2^ when administered at 1 × 10^8^ MB/mL. In this study, MBs were administered at 4 × 10^8^ MB/mL, which after allowing for flotation is MB density of equivalent to 1.9 × 10^5^ MB/mm^2^, an equivalent increase of ∼30 × . As such, future work should investigate the influence of both un-targeted and targeted MB concentration of biofilm dispersal.

The biomass stain SYTO9 was used throughout this study to determine the presence of biofilm biomass, which alone is unable to determine viability of bacteria present in the biofilm. It would be expected that any US + MB induced kill of bacteria would be due to compromise of the bacterial cell membrane via sonoporation. In a study of sonoporation of individual *S. aureus* bacterium in a biofilm, Lattwein et al. [29] found that a maximum of only 2.5 % of cells experienced an increase in membrane permeability (PNP = 400 kPa), whilst recent work from our group found a modest increase of 8 % in dead cell count (PNP = 800 kPa) after treatment with US + MB. It should be noted that these studies utilized SYTO9 combined with propidium iodide to determine bacterial viability, which has been attributed to an underestimation in bacterial cell viability, potentially due to the presence of extracellular nucleic acids [77,78].

Further, these studies and the current study fail to assess the fate of dispersed bacteria. This is of clinical importance as treatment with US + MB may simply disperse viable bacteria from a surface and spread the infection. Childers et al. [31] found that in a catheter infection model of *S. aureus*, treatment with US + MBs could only achieve a 0.26 ± 0.26 log10 reduction in colony forming units removed from the catheter lumen, despite the use of a high-power histotripsy system utilizing a PNP of 12.3 MPa.

Hence, in our study it is unlikely that a significant proportion of bacteria, dispersed or not, experienced sonoporation, or a subsequent change in viability. Predicted shear stresses produced by a MB undergoing stable oscillation or inertial cavitation are in the range of 1–20 kPa and 30–700 kPa [79], respectively. Further, in a sonoporation study of mammalian cells Elburg et al. speculated that the normal stress was the dominant mechanism behind sonoporation, achieving stresses 2 to 3 orders of magnitude higher than wall shear stress [80]. To the best of our knowledge, there are currently no studies investigating the threshold shear stress needed to effect viability of S. aureus (gram-positive), whilst data for E. coli (gram-negative) cover a broad range (10^3^–10^7^ Pa [81,82]) and are likely to be lower than required for gram-positive species.

Combining US + MB mediated biofilm dispersal with antimicrobial agents may enhance treatment efficacy whilst reducing the risk of spreading viable bacteria. In its simplest form, this approach could involve co-delivery of US + MBs alongside “free” antibiotics. The MB-induced disruption of the biofilm may improve antibiotic penetration and effectiveness, potentially allowing for a reduced total antibiotic dose. This approach could be particularly effective in an antibiotic lock setting, where the lumen of an indwelling catheter is filled with an antibiotic to treat and prevent catheter-related infections, ultimately aiming for catheter salvage [83]. Current guidance recommends antibiotic concentrations exceeding 1000 times the MIC and a treatment duration of two weeks [84], during which the catheter is non-functional. By integrating MB-mediated biofilm disruption, it may be possible to lower both the required antibiotic concentration and treatment duration.

To further reduce the risk of biofilm dispersal and subsequent recolonisation, the antibiotic-MB solution could be withdrawn following treatment. There may also be optimal US conditions and MB concentration to allow for sufficient drug penetration into the biofilm, whilst leading to minimal dispersal of viable bacteria.

Additionally, antimicrobials could be incorporated directly into MBs—either within the microbubble shell [8,9] or via attachment of drug-loaded liposomes [10,11]. In this case, US could be used to trigger the targeted release of the antimicrobial payload at the infection site, helping to reduce systemic drug exposure.

For successful clinical translation, the choice and complexity of the ultrasound system to perform the treatment needs to be considered. One possibility could be through the use of a diagnostic ultrasound system equipped with contrast-enhanced ultrasound (CEUS) capabilities. In this context, the system can image microbubbles (MBs) exclusively through non-linear imaging modes. These systems often include a high-pressure “flash” mode, primarily designed to destroy MBs and observe their refilling dynamics. The “flash” mode would serve as an alternative to the PNP = 2500 kPa MB destruction condition. Here, the clinician would be able to manually translate the transducer along the infection site, visualising the presence of MBs within the image region before initiating the “flash” function to trigger MB destruction.

A more advanced but robust alternative is image-guided HIFU, already used in a clinical setting for treating conditions such as prostate cancer [85], uterine fibroids [86] and essential tremor [87]. These systems use real-time imaging (ultrasound or MRI) to target tissue and monitor treatment progress. A similar approach could be adapted to treat infections along catheters or other implants, allowing for precise, localised ultrasound therapy. Unlike traditional HIFU, which relies on thermal ablation, our method uses MBs as cavitation nuclei, allowing lower peak negative pressure, duty cycle, and treatment duration and hence minimising thermal effects. We also note that the MBs used in this study follow a similar formulation and size distribution to those used in the clinic [49,88], further aiding translation.

The use of targeted MBs also opens an additional clinical application, in which targeted MBs could be used to locate the biofilm via diagnostic ultrasound imaging. These MBs could then be destroyed through a high-pressure US pulse, with the MB destruction confirmed via subsequent imaging. Successful removal of the biofilm could be further validated through a subsequent round of targeted microbubble (MB) infusion, followed by ultrasound imaging to assess the presence or reduction of MB-biofilm attachment. While these techniques are feasible in a clinical setting, dedicated studies would be required to demonstrate their efficacy—an investigation that falls beyond the scope of this work.

## 5 Conclusion

In summary, we have demonstrated the ability of US + MBs to nearly totally disperse *S. aureus* biofilms cultured on the surface of a microfluidic device. We compared the biofilm dispersal capabilities at two different ultrasound pressures: 360 kPa and 2500 kPa, as well as the influence of the direction of the applied US (i.e. acoustic radiation force pushing MBs towards, US↑, or away, US↓, from the biofilm). For US↑ + MB, a PNP of 360 kPa resulted in minimal biofilm removal. Increasing PNP to 2500 kPa caused near total biofilm dispersal within the focal region of the US beam (94 ± 2 %). Changing US direction to US↓ significantly reduced biofilm removal at 2500 kPa (∗∗, p < 0.01), demonstrating the importance of the direction of the acoustic radiation force in biofilm removal. However, large amounts of biofilm could still be dispersed within the US focal region (81 ± 3 %). Biofilm morphology could be controlled by pre-treating the growth surface with either fibrinogen or human plasma, increasing both biofilm roughness and thickness. Regardless, >85 % of biofilm could still be dispersed by US + MB treatment. Multiple US + MB treatments could be used to nearly totally disperse biofilm across a larger total area, and that biofilm could still be removed either with or without MB replenishment between each US treatment. Closer observation of MB behaviour suggested that movement of oscillating MBs through the biofilm may be the main mechanism behind biofilm dispersal, and that the larger MBs account for the majority of observed biofilm removal.

## CRediT authorship contribution statement

**Damien V.B. Batchelor:** Writing – review & editing, Writing – original draft, Validation, Software, Methodology, Investigation, Formal analysis, Conceptualization. **Anjali Lad:** Writing – review & editing. **Kathryn L. Burr:** Writing – review & editing. **Kristian Hollie:** Writing – review & editing. **James R. McLaughlan:** Writing – review & editing, Resources. **W. Bruce Turnbull:** Writing – review & editing, Supervision, Funding acquisition, Conceptualization. **Jonathan A.T. Sandoe:** Writing – review & editing, Supervision, Funding acquisition, Conceptualization. **Stephen D. Evans:** Writing – review & editing, Supervision, Resources, Funding acquisition, Conceptualization.

## Declaration of competing interest

The authors declare the following financial interests/personal relationships which may be considered as potential competing interests: Prof. Stephen Evans reports financial support was provided by Engineering and Physical Sciences Research Council. Prof. Stephen Evans reports financial support was provided by 10.13039/100006662National Institute for Health and Care Research (NIHR) Leeds 10.13039/100005515Biomedical Research Centre (BRC) (NIHR213331). If there are other authors, they declare that they have no known competing financial interests or personal relationships that could have appeared to influence the work reported in this paper.

## Data Availability

Data will be available at https://doi.org/10.5518/1727 after publication.
