# Supplementary material for: S. aureus biofilm disruption using ultrasound and microbubbles: Influence of radiation force, bubble dynamics and biofilm growth conditions
Source: Biofilm. 2025 Oct 25;10:100327. doi: 10.1016/j.bioflm.2025.100327 (PMC12648606; doi:10.1016/j.bioflm.2025.100327)
Supplement: Multimedia component 1 [file mmc1.docx]

**Supplementary Material: *S. aureus* Biofilm Disruption Using Ultrasound and Microbubbles: Influence of Radiation Force, Bubble Dynamics and Biofilm Growth Conditions**

Damien V. B. Batchelor ^1^, Anjali Lad^1^, Kathryn L. Burr ^2^, Kristian Hollie ^2^, James R. McLaughlan^3,4^, W. Bruce Turnbull ^2^, Jonathan A. T. Sandoe ^5^ and Stephen D. Evans* ^1^

*^1^School of Physics and Astronomy, University of Leeds, Leeds, UK, LS2 9JT*

*^2^School of Chemistry and Astbury Centre for Structural Molecular Biology, University of Leeds, Leeds, UK, LS2 9JT*

*^3^School of Electronic and Electrical Engineering, University of Leeds, Leeds, UK, LS2 9JT*

*^4^Leeds Institute of Medical Research, University of Leeds, St James’ University Hospital, Leeds, LS9 7TF, UK*

*^5^School of Medicine and Health, University of Leeds, Leeds, UK, LS2 9JT*

**Corresponding Author*

*Email:* [*S.D.Evans@leeds.ac.uk*](mailto:S.D.Evans@leeds.ac.uk)*,*

| *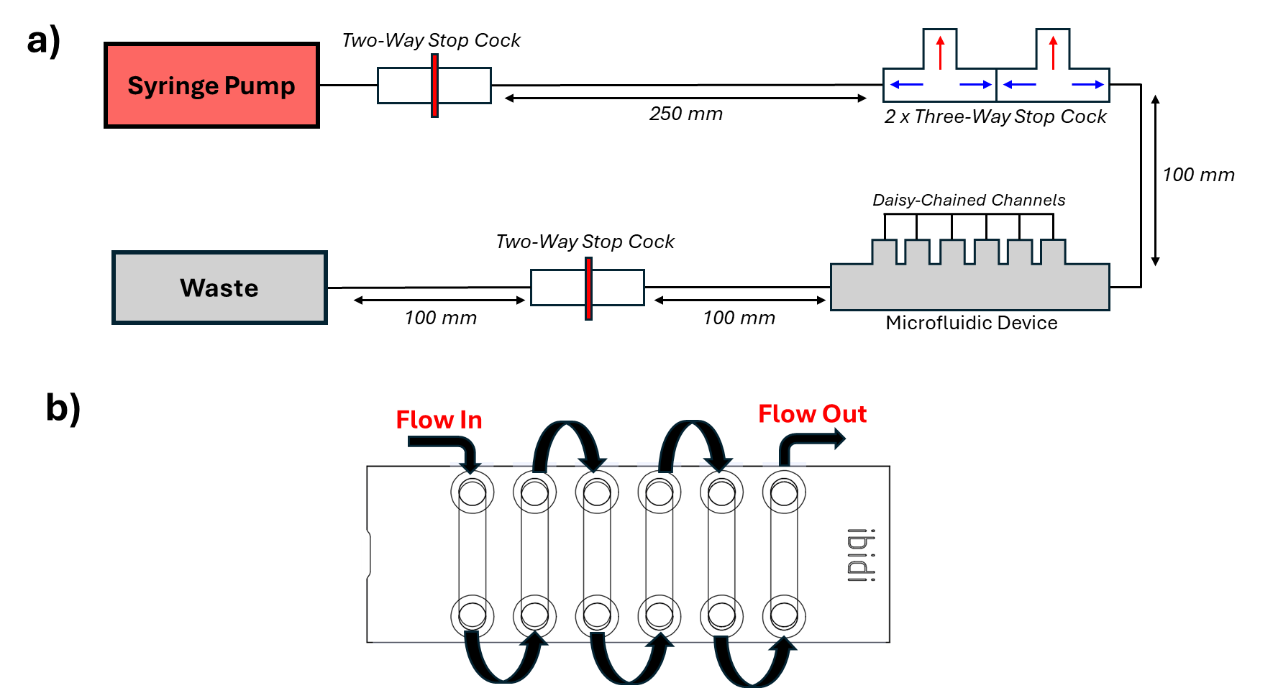* |
| --- |
| **Figure S1 ­–** (a) Schematic showing the fluidic experimental set-up for microfluidic growth of S. aureus biofilms. Flow was controlled by a syringe and syringe pump. The system was inoculated by manual injection of 5 mL of a bacterial solution (0.5 × 10^8^ CFU/mL) through the first 3-way stopcock. The presence of the 2^nd^ stopcock allowed the removal of any unwanted air bubbles introduced into the system at this stage. (b) Schematic showing daisy-chained connections between neighbouring channels. |

| *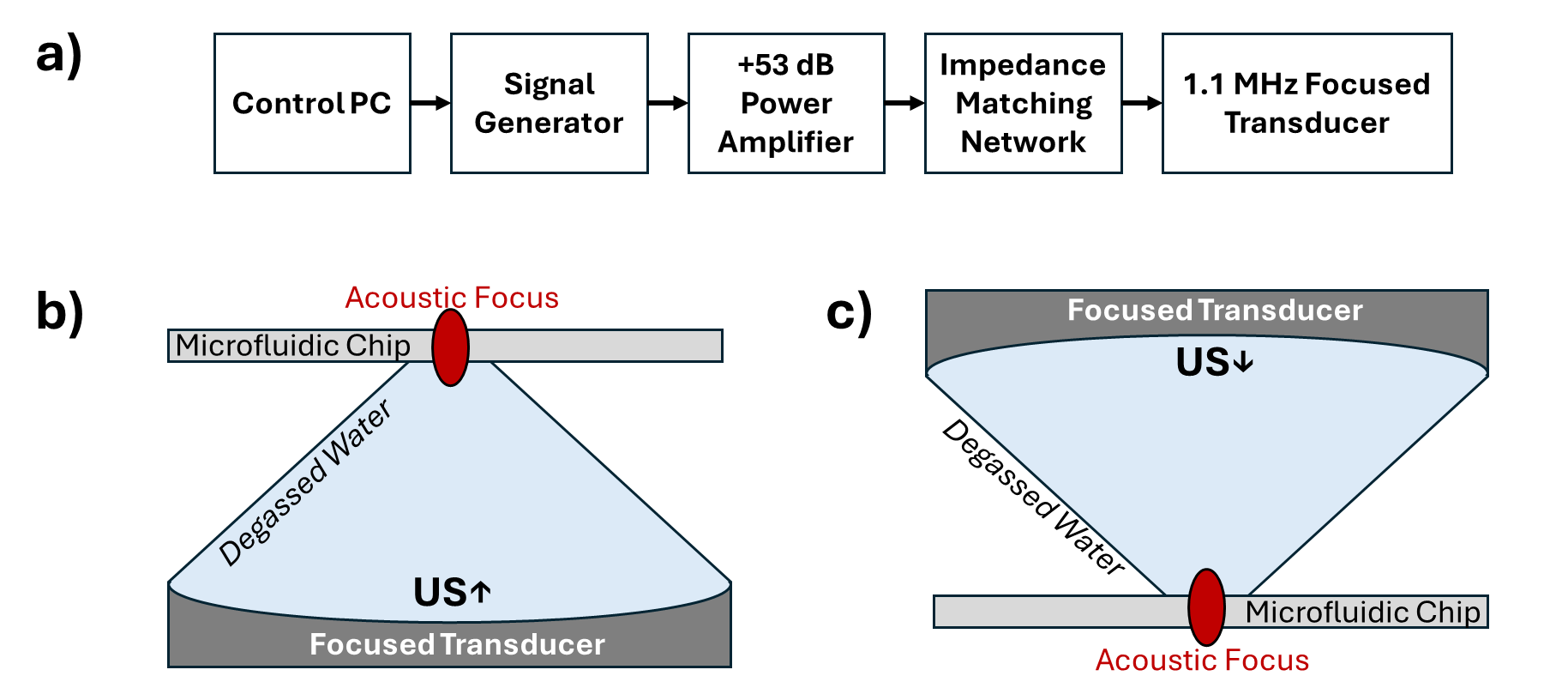* |
| --- |
| **Figure S2** –a) Diagram of connection of components of the ultrasound system used for US treatment of biofilms. Schematics showing orientation of transducer for (b) US↑ and (c) US↓ with the position of acoustic focus shown in red. |

| **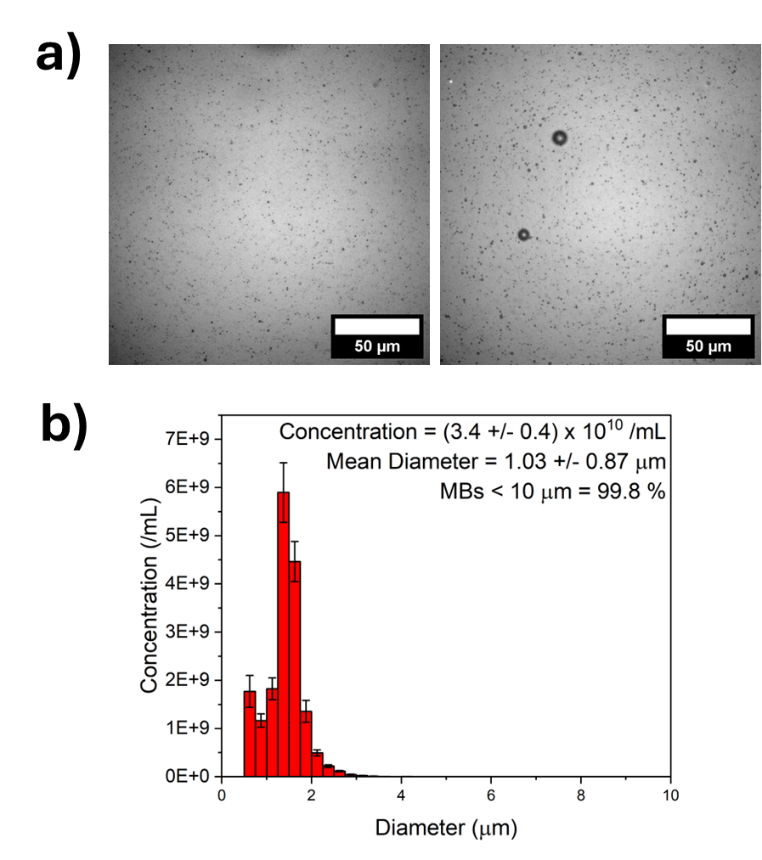** |
| --- |
| \| *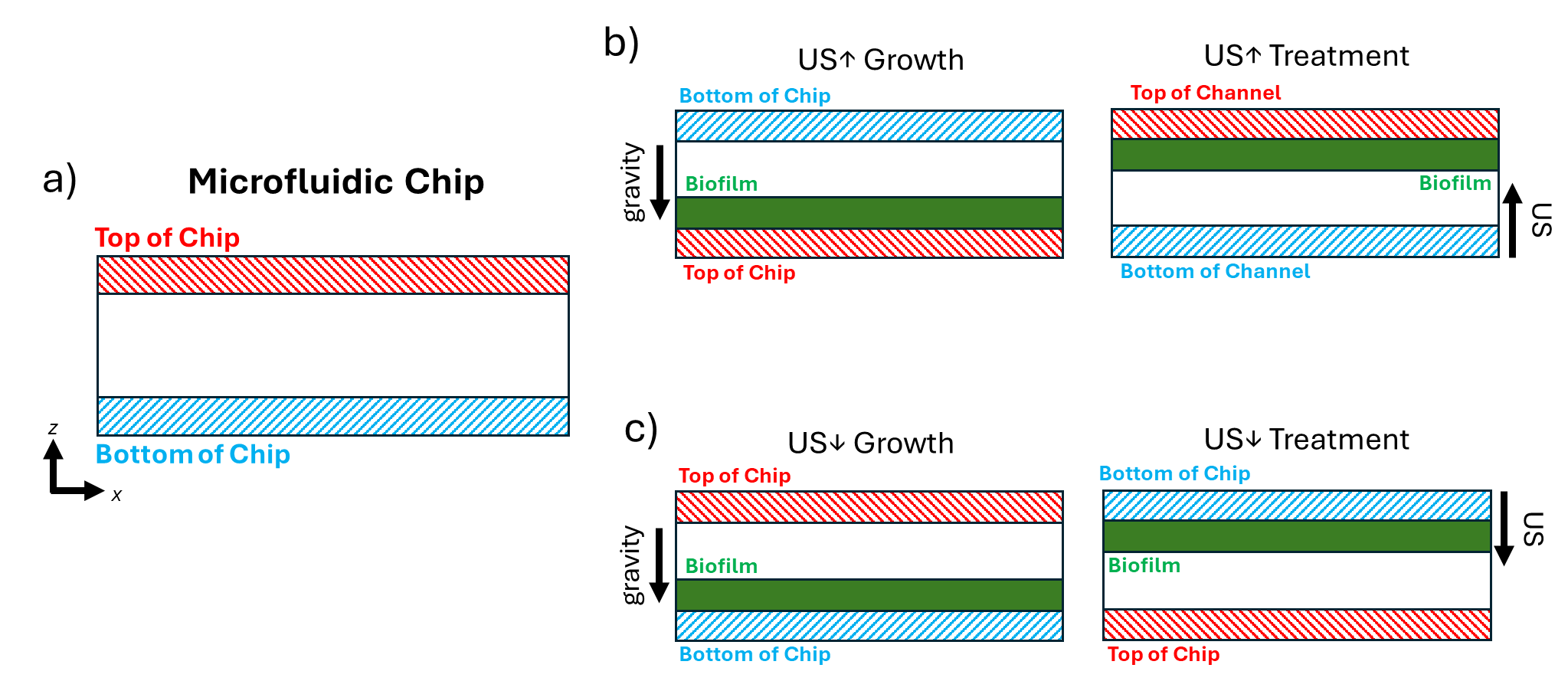* \| \| --- \| \| **Figure S4:** Schematic showing the biofilm growth and ultrasound (US) treatment conditions within the microfluidic chip, denoting the top and bottom of the chip. a) Schematic of microfluidic chip in its upright position. Orientation of microfluidic chip for biofilm growth and treatment for (b) US↑ and (c) US↓. \|   **Figure S3:** a) Brightfield microscopy of MBs produced by mechanical agitation. MBs were diluted 50 x prior to imaging. b) Corresponding size distribution, averaged over all MB samples used in this study. |
| **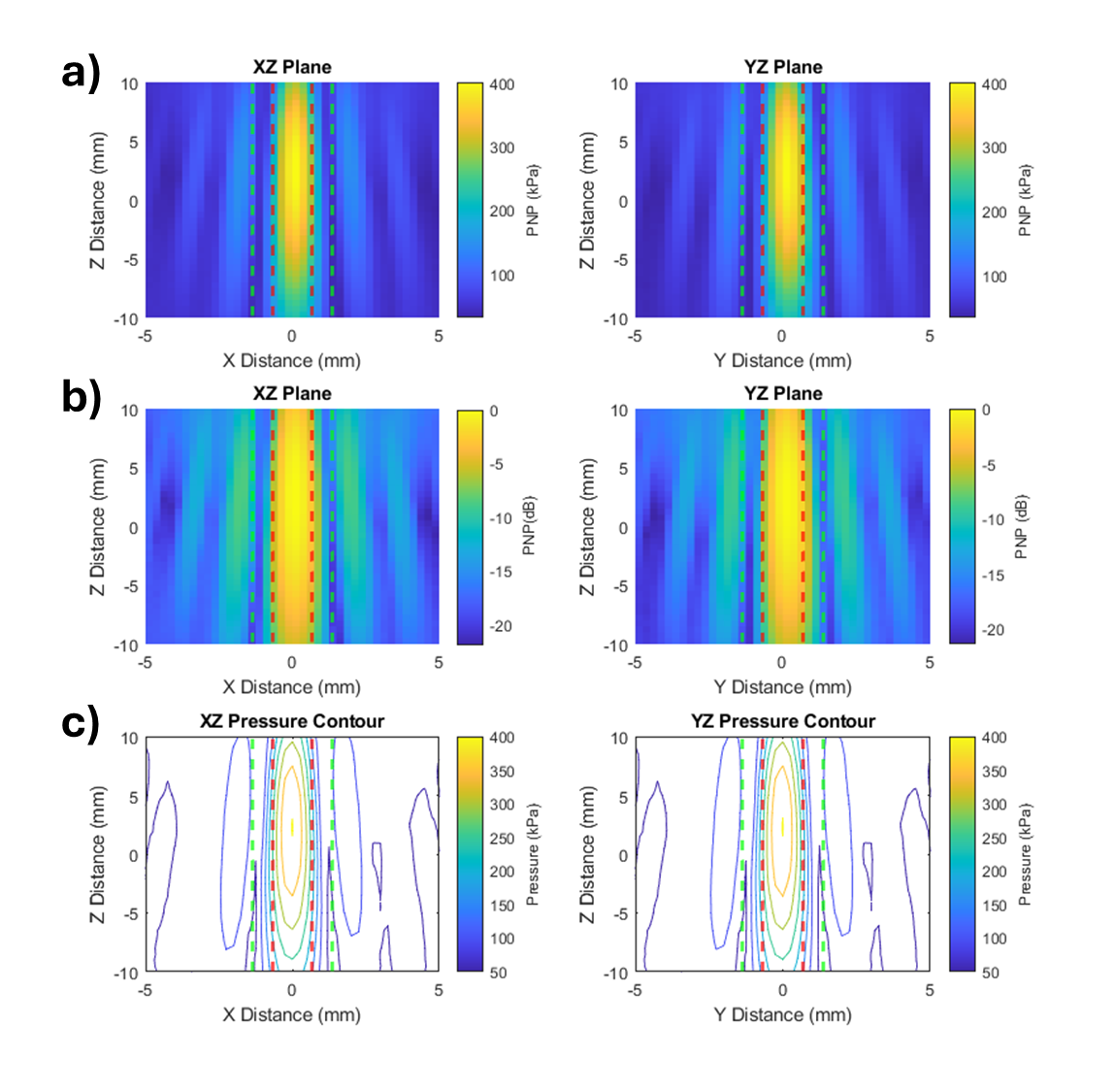** |
| **Figure S5***: HIFU US transducer beam plot measured for a PNP of 400 kPa in both the XZ and YZ planes. a) PNP in kPa. b) PNP measured in decibels (dB) relative to maximum PNP value. c) Contour plot showing gradient of PNP in kPa. Red dashed line: R_US._ Green dashed line: 2R_US_.* |

| **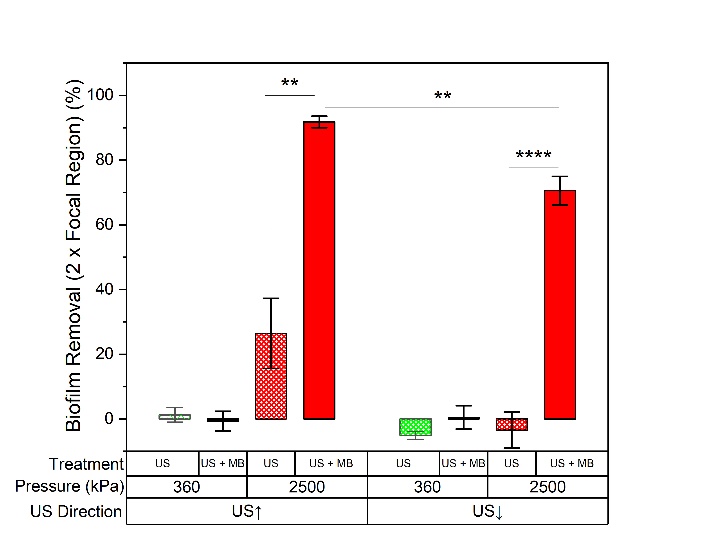** |
| --- |
| **Figure S6**: *Total biofilm removal within 2R_US_ for US↑ and US↓ at a PNP of either 360 kPa or 2500 kPa, for both US only and US + MBs conditions.* |

| 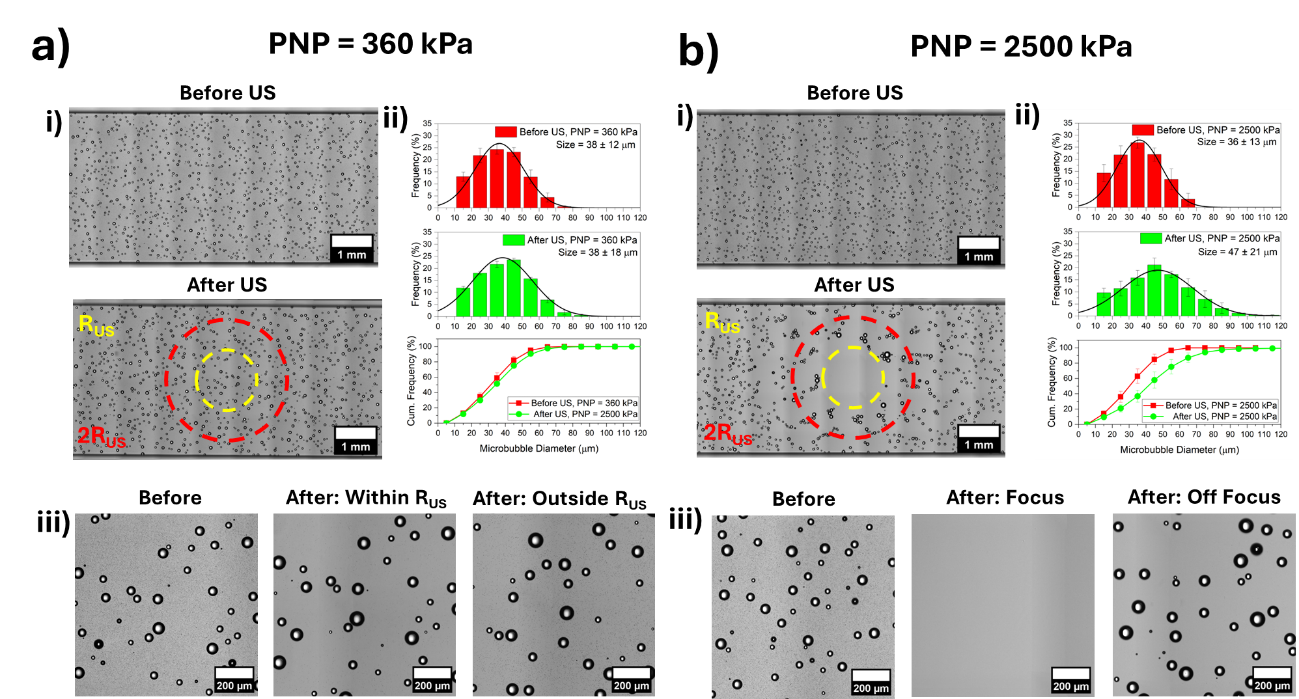 |
| --- |
| **Figure S7***: Transmission microscopy images of MBs on-chip before and after treatment US. Here, only large MBs (> 5 um) (a) PNP = 360 kPa and (b) PNP = 2500 kPa. i) Images of the entire microfluidic chip before and after US treatment. Yellow dashed circle: focal area of the US beam with a radius R_US_. Red dashed circle: 2R_US._ ii) Size and cumulative frequency distribution of large MBs before and after US treatment.* |

| 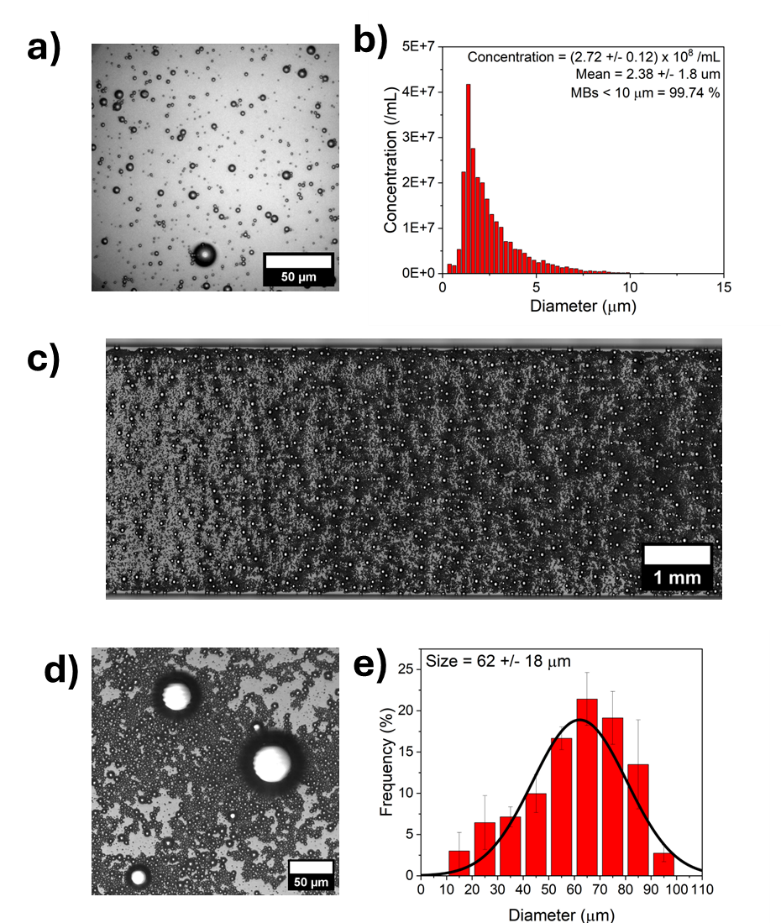 |
| --- |
| **Figure S8***:* *Microscopy images of SonoVue microbubbles. A) Brightfield microscopy used for characterisation of MB size and concentration and (b) the corresponding population distribution. C) TileScan image of SonoVue MBs on-chip. d) Increased magnification image of SonoVue MBs on-chip. e) Population distribution taken from TileScan images showing population of larger SonoVue MBs.*  **Method S1**: To determine background intensity of MB images (Figure S6, S8), a binary mask was generated to remove the presence of any large MBs from the image, leaving behind just the background due to small MBs. The background intensity was then determined as a function of horizontal distance from the centre of the chip ${(I}_{\mathrm{treated}}\left( x \right))$. This was then normalised as a percentage change to the original mean intensity of the image containing MBs only, $I_{\mathrm{untreated}}$.  $Change in Background Intensity \left( \% \right)=100 x \left( \frac{I_{\mathrm{treated}}\left( x \right)-\bar{I_{\mathrm{untreated}}}}{\bar{I_{\mathrm{untreated}}}} \right)$ |
| 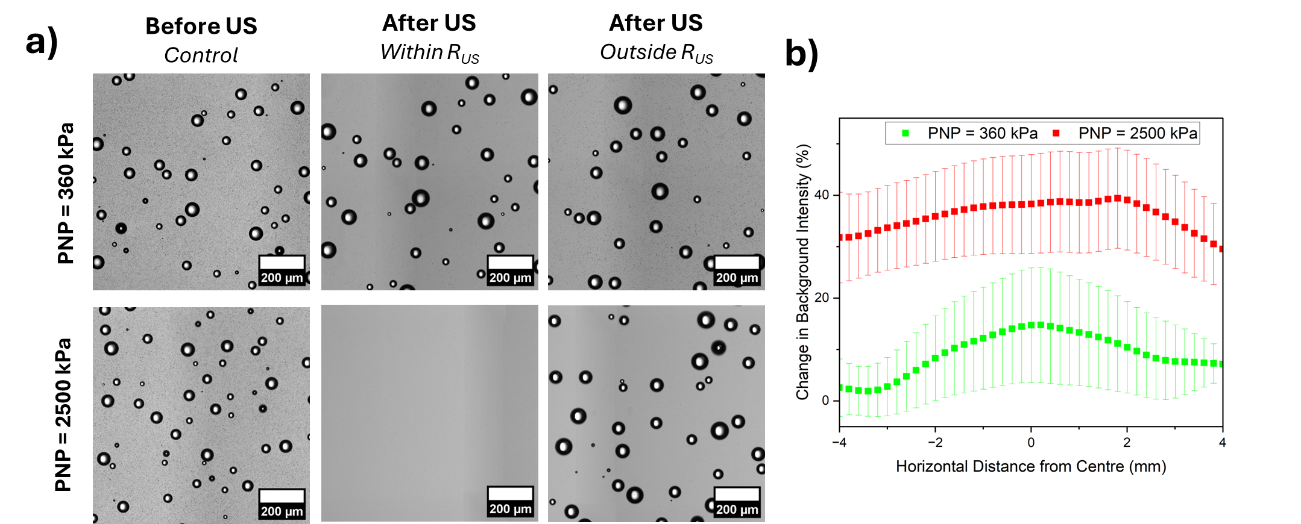 |
| **Figure S9***:* *a) Higher magnification images of MBs before and after US, showing areas both within the US focal region (within R_US_) and outside of US focal region (outside R_US_). Large MBs are clearly visible whereas the presence of small MBs is shown by presence of small speckles in the background of the image. c) Change in image background intensity of images in (a) as a function of x-distance, after application of US at PNP = 360 kPa or 2500 kPa, used as a metric to determine destruction of small MBs.* |

| 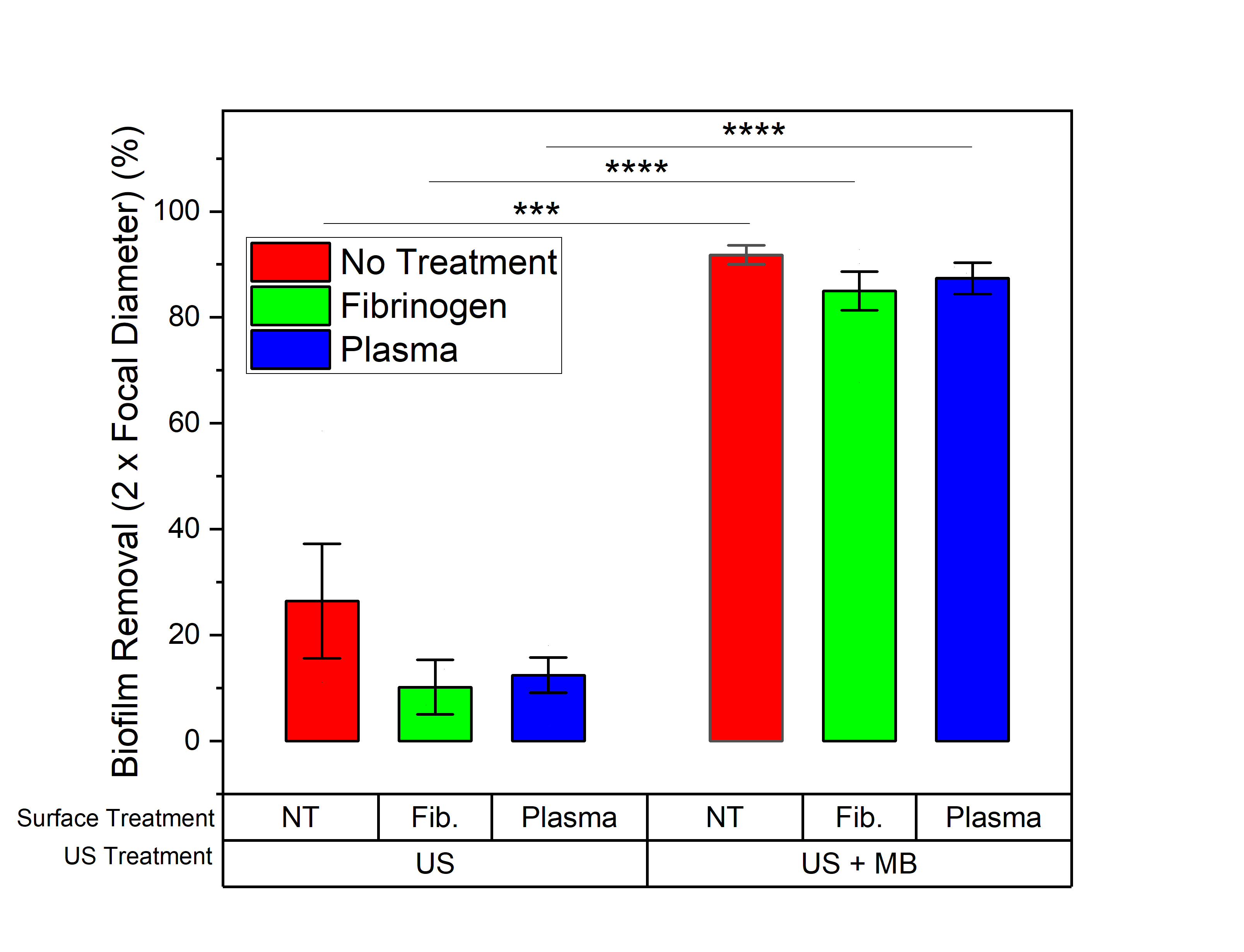 |
| --- |
| **Figure S10**: Total biofilm removal within 2R_US_ of the US beam after treatment with US or US + MB (PNP = 2500 kPa) for biofilms grown on surfaces with no pre-treatment (NT) or treatment with Fibrinogen (Fib.) or human plasma. |

| 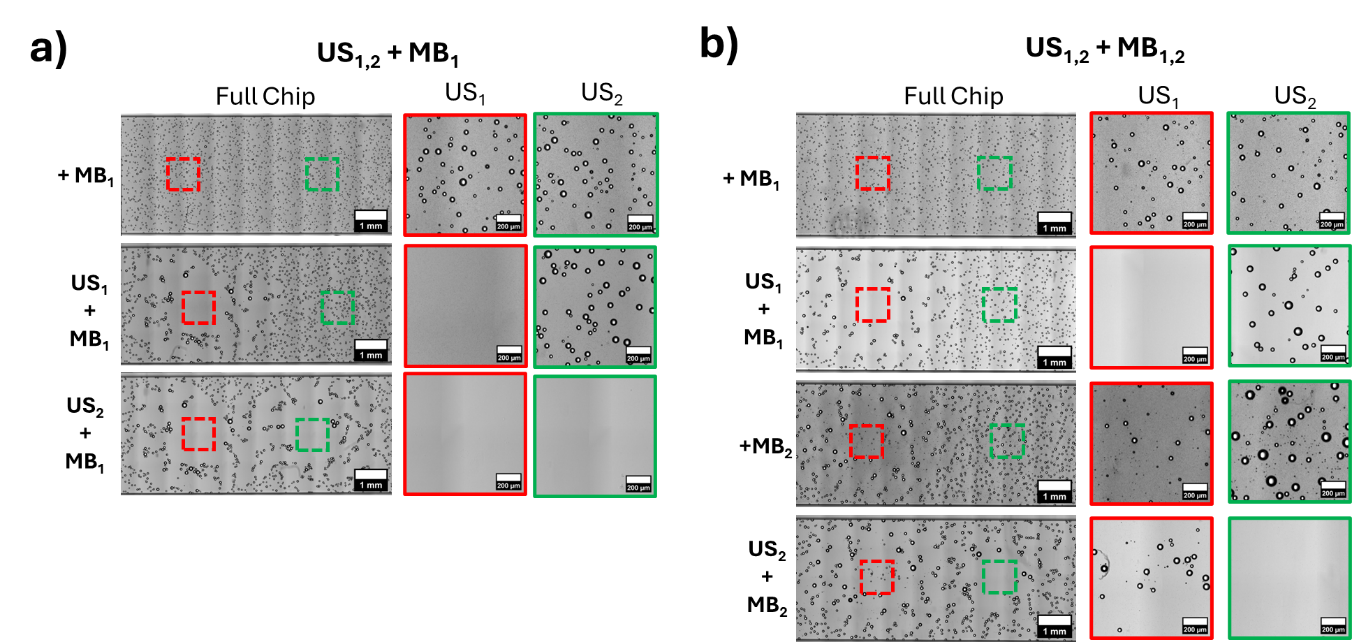 |
| --- |
| **Figure S11:** Transmission microscopy images of microfluidic chips for each stage of (a) US_1,2_ + MB_1_ (no MB refill between US treatments) and (b) US_1,2_ MB_1,2_ (MB refill between each US treatment). TileScan images of the entire microfluidic chip were taken after each addition of MBs or treatment with US. Areas within the region for each US treatment (US1, US_2_) are highlighted by red and green borders respectively. |

| 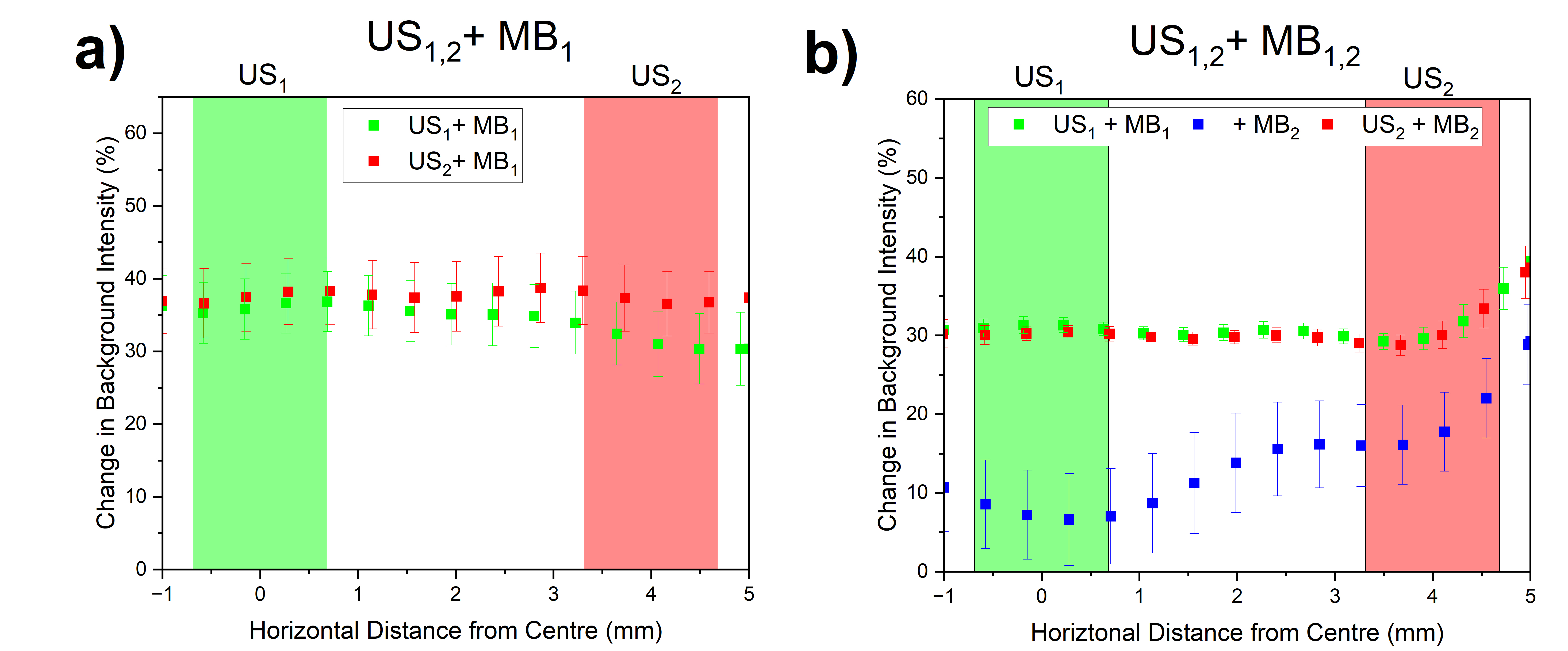 |
| --- |
| **Figure S12:** Change in background intensity of images shown in Figure S11, used as a metric of small MB concentration, as a function of distance from the centre of US_1_ for (a) US_1,2_ + MB_1_ and (b) US_1,2_ MB_1,2_ treatment conditions, after each treatment stage. Focal area for US­_1_ is shown as a shaded green box and for US_2_ as a shaded red box. |

**Methods S2**

Peak acoustic intensity of an ultrasound beam, I_peak_, can be determined used Equation 1[1], where P is peak negative pressure ρ is density of water (1000 kg/m^3^) and c is speed of sound in water (1480 m/s). Hence, for P = 2500 kPa, I_peak_ = 211 W/cm^2^.

|  | $I_{peak}=P^{2}/\rho c$ | Equation 1 |
| --- | --- | --- |

[1] S. Murthi, M. Ferguson, and A. Sisely, “Diagnostic Ultrasound - physics and equipment,” in *Diagnostic Ultrasound - physics and equipment*, 2nd ed., 2010, ch. 2, pp. 4–22.
